# Retinal microglia protect against vascular damage in a mouse model of retinopathy of prematurity

**DOI:** 10.3389/fphar.2022.945130

**Published:** 2022-08-17

**Authors:** Jin Liu, Jessica Kwan Wun Tsang, Frederic Khe Cheong Fung, Sookja Kim Chung, Zhongjie Fu, Amy Cheuk Yin Lo

**Affiliations:** ^1^ Department of Ophthalmology, School of Clinical Medicine, Li Ka Shing Faculty of Medicine, The University of Hong Kong, Hong Kong, Hong Kong SAR, China; ^2^ School of Biomedical Sciences, Li Ka Shing Faculty of Medicine, The University of Hong Kong, Hong Kong, Hong Kong SAR, China; ^3^ Department of Ophthalmology, Boston Children’s Hospital, Harvard Medical School, Boston, MA, United States

**Keywords:** animal models, blindness, endothelial cell, eye, inflammation, neonate, preterm, vascular protection

## Abstract

Retinopathy of prematurity (ROP) is a common cause of blindness in preterm babies. As a hypoxia-induced eye disease characterized by neovascularization, its association with retinal microglia has been noted but not well documented. We performed a comprehensive analysis of retinal microglia and retinal vessels in mouse oxygen-induced retinopathy (OIR), an animal model of ROP. In combination with a pharmacological inhibitory strategy, the role of retinal microglia in vascular network maintenance was investigated. Postnatal day (P) 7 C57BL/6J mouse pups with their nursing mother were exposed to 75% oxygen for 5 days to induce OIR. Age-matched room air-treated pups served as controls. On P12, P17, P21, P25, and P30, retinal microglia and vessels were visualized and quantified based on their location and activation status. Their relationship with retinal vessels was also analyzed. On P5 or P12, retinal microglia inhibition was achieved by intravitreal injection of liposomes containing clodronate (CLD); retinal vasculature and microglia were examined in P12 and P17 OIR retinae. The number of retinal microglia was increased in the superficial areas of OIR retinae on P12, P17, P21, P25, and P30, and most of them displayed an amoeboid (activated) morphology. The increased retinal microglia were associated with increased superficial retinal vessels in OIR retinae. The number of retinal microglia in deep retinal areas of OIR retinae also increased from P17 to P30 with a ramified morphology, which was not associated with reduced retinal vessels. Intravitreal injection of liposomes-CLD caused a significant reduction in retinal microglia. Loss of retinal microglia before hyperoxia treatment resulted in increased vessel obliteration on P12 and subsequent neovascularization on P17 in OIR retinae. Meanwhile, loss of retinal microglia immediately after hyperoxia treatment on P12 also led to more neovascularization in P17 OIR retinae. Our data showed that activated microglia were strongly associated with vascular abnormalities upon OIR. Retinal microglial activation continued throughout OIR and lasted until after retinal vessel recovery. Pharmacological inhibition of retinal microglia in either hyperoxic or hypoxic stage of OIR exacerbated retinal vascular consequences. These results suggested that retinal microglia may play a protective role in retinal vasculature maintenance in the OIR process.

## 1 Introduction

Retinopathy of prematurity (ROP), a common complication of oxygen therapy in preterm infants, is a major cause of avoidable blindness among children in established market economies and is emerging in middle-income countries nowadays (Gilbert and Foster, 2001). Mild and moderate ROP usually recovers spontaneously, while advanced ROP needs therapeutic intervention. Current standard treatments for advanced ROP are retina ablative therapy using laser or cryotherapy; however, they sacrifice part of vision to stop ROP. Clinical application of anti-vascular endothelial growth factor (VEGF) therapy has been used to treat proliferative ROP. However, as VEGF also plays important physiological functions during infant development, simply blocking VEGF may have a negative influence on brain, lung, and kidney development as well as retina health (Mintz-Hittner et al., 2011; Autrata et al., 2012; Wu et al., 2013). Effective preventive strategies against ROP with fewer side effects are warranted.

Mouse oxygen-induced retinopathy (OIR) is a well-established and widely accepted rodent model that mimics ROP (Connor et al., 2009; Stahl et al., 2010b), which is used in studying the pathological mechanisms of ROP and other vascular proliferative pathologies. In the mouse OIR model, central vaso-obliteration induced by hyperoxia and neovascularizations (NVs) caused by relative hypoxia in a room air (RA) environment mimics phase I and phase II of ROP, respectively.

Microglia are a unique immune cell population in the central nervous system (CNS) and considered macrophages in the CNS (Kettenmann et al., 2011). Microglia originate from progenitor cells entering CNS via primitive bloodstream from yolk sac on embryonic (E) day 8.5–9.0 (Salter and Beggs, 2014). In the mouse retina, microglia first appear on E11.5 in the central part of the retina and then migrate tangentially and radially till they cover all retinal layers except the outer nuclear layer (ONL) by about postnatal day (P) 7 (Santos et al., 2008). These cells function as safeguards, maintaining homeostasis of the local microenvironment while participating in modulation of synaptic plasticity (Salter and Beggs, 2014). Under normal conditions, these cells display a ramified morphology; once activated, microglia transfer to an amoeboid morphology (Kettenmann et al., 2011). Retinal microglia also participate in retinal vasculature development, influencing the early development and refinement of retinal vasculature (Arnold and Betsholtz, 2013). Depletion of retinal microglia resulted in a delayed retinal vascular development (Checchin et al., 2006).

Retinal microglia have been noted to be involved in the OIR model while their role in the OIR process is still unclear. Retinal microglia, rather than infiltrating blood-derived macrophages, are the predominant myeloid cells in the NV area in mouse OIR (Boeck et al., 2020). Retinal microglia were observed to be increased in OIR retinal sections, located in areas with NVs on P17 and associated with avascular outer retinal layers on P21 (Davies et al., 2006). Vessey et al. (2011) found that in P18 OIR retinal sections, the number of retinal microglia was increased, and it was associated with a loss of deep retinal vessels. We previously reported retinal microglia activation in P17 OIR retinae (Fu et al., 2012). Intravitreal injection of myeloid progenitor cells could ameliorate NVs in the mouse OIR model, and these injected progenitor cells finally differentiated into microglia-like cells (Ritter et al., 2006). Further study showed that microglia might cooperate with astrocytes, promote the regrowth of blood vessels after vaso-obliteration in OIR, and thus result in less NVs afterward in the mouse OIR model via VEGF and bFGF (Dorrell et al., 2010). In a rat OIR model, inhibition of microglia led to suppressed neovascularization tufts, suggesting that those monocytes might be harmful to retinal vasculature during OIR (Ishida et al., 2003). CX3C chemokine receptor 1 (CX3CR1) is essential for monocyte recruitment and thus essential for the proper functioning of monocytes, such as macrophages/microglia. However, CX3CR1-deficient mice showed unaffected vaso-obliteration or NVs in the OIR model (Liang et al., 2009). The role of microglia in OIR pathogenesis is still controversial; it is difficult to conclude whether microglia are beneficial or detrimental for retinal vasculature in the OIR model based on current experimental evidence.

Clodronate is a first-generation bisphosphonate for treatment of osteoporosis (Muratore et al., 2011); it is also used to deplete macrophages/microglia specifically. Clodronate is unstable in the circulation; when encapsulated in liposomes, clodronate can then exist in the circulation. Furthermore, these liposomes can be solely engulfed by macrophages/microglia due to their phagocytic properties; therefore, clodronate can specifically enter macrophages/microglia (van Rooijen and Sanders, 1994). Once present in the cytoplasm of macrophages/microglia, clodronate triggers apoptosis, probably due to competitive inhibition of the ATP/ADP translocase by its metabolite adenosine-5'-[beta,gamma-dichloromethylene]triphosphate (AppCCl(2)p) that inhibits mitochondrial oxygen consumption (Lehenkari et al., 2002). This process is called “macrophage suicide” and is currently widely used and accepted for the depletion of macrophages (Hitchcock et al., 2015; Masuch et al., 2016; Ngoh et al., 2016).

In the present study, we investigated retinal microglia behavior in a mouse OIR model in a detailed manner, with their quantity, activation status, and location clarified at various time points during the OIR challenge. Meanwhile, their relationship with retinal vessels was also examined. Furthermore, liposomes-clodronate was intravitreally injected to inhibit microglial function before or after hyperoxia treatment of OIR so that the role of microglia at different stages of OIR was further evaluated.

## 2 Materials and methods

### 2.1 Animals

Normal C57BL/6J mice used in the present study were obtained from The Jackson Laboratory (#000664) and housed in a temperature-controlled room under a 12-h light/dark cycle provided by fluorescent light in recessed lighting consoles. They were allowed free access to food and water in the Laboratory Animal Unit of The University of Hong Kong. All the experimental and animal handling procedures were in accordance with the ARVO Statement for the Use of Animals in Ophthalmic and Vision Research. The use of animals was conducted according to the requirements of the Cap. 340 Animals (Control of Experiments) Ordinance and Regulations, and all relevant legislation and Codes of Practice in Hong Kong and was approved by the Faulty Committee on the Use of Live Animals in Teaching and Research in The University of Hong Kong (CULATR #3121–13 and #3464–14).

### 2.2 Animal model of OIR

**FIGURE 1:**
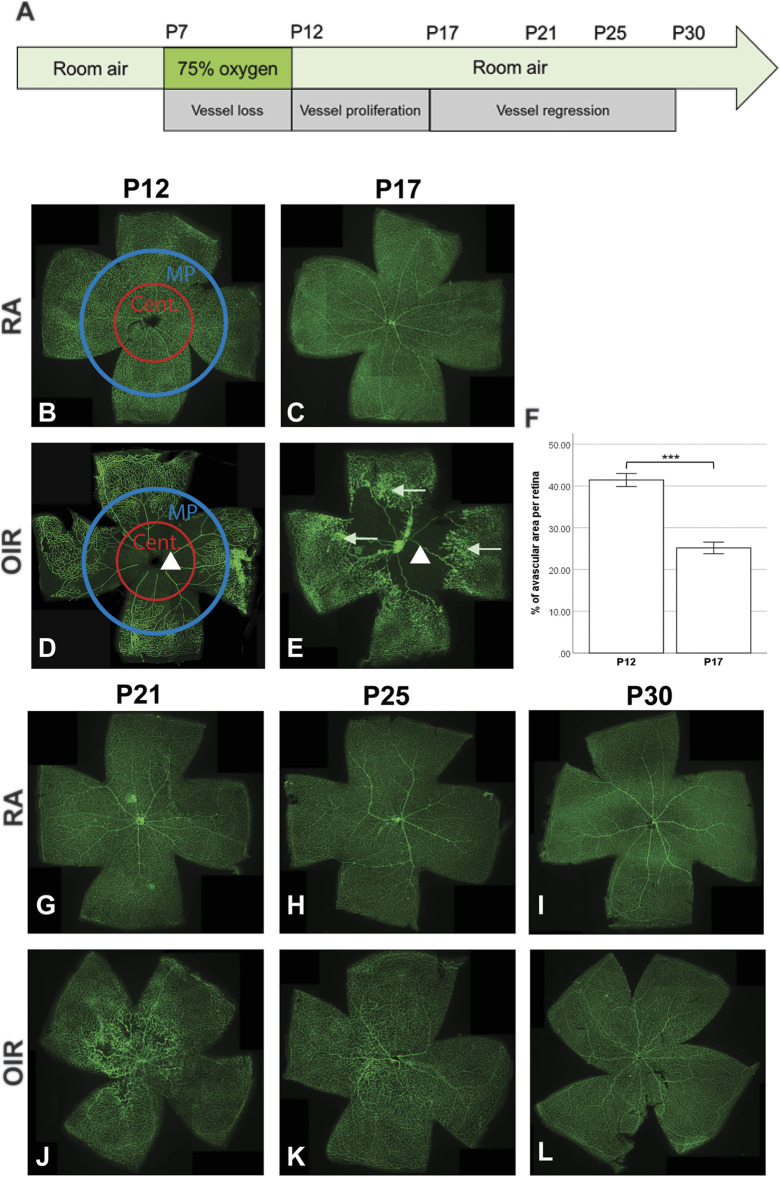
Retinal vasculature changes during the OIR process. Representative pictures of retinal vasculature of RA and OIR retinae are shown. Schematic diagram indicating the mouse OIR model **(A)**. RA retinae showed normal retinal vessel development with no obvious abnormalities **(B,C,G,H,I)**. Central (Cent.) and mid-peripheral (MP) areas were indicated in P12 RA and OIR retinae **(B,D)**. P12 OIR retinae showed a central avascular zone (indicated by the triangle in **(D)**. The central avascular zone could still be observed in P17 OIR retinae (indicated by the triangle in **(E)**), but the size of the avascular area was decreased, compared with that in P12 OIR retinae **(F)**. Neovascularizations (indicated by arrows in **(E)**) could be observed in the mid-peripheral sites of P17 OIR retinae. From P21 to P30, the neovascularizations regressed, and the OIR retinae were fully covered by retinal vessels **(J**–**L)**. n = 6–8 in each group, unpaired *t*-test, ***p < 0.001.

The establishment of the mouse OIR model was performed as previously described with modifications (Smith et al., 1994; Fu et al., 2012). Briefly, neonatal mouse pups and their nursing mothers were placed in an air-tight chamber with 75% oxygen (PRO-OX110 chamber controller; Biospherix Ltd., New York, NY) from P7 to P12 and then returned to the room air 21% oxygen environment on P12 (Figure 1A). Soda lime (Sigma-Aldrich Corporation, St. Louis, MO) was placed in the chamber during the high oxygen treatment to quench excess CO_2_. Activated carbon and silica gel desiccants were also placed in the chamber for ammonia and humidity control, respectively. The litter size was restricted to 6 to 7 pups in each litter to make sure all pups in the cage received enough nutrition (Connor et al., 2009). Retina samples were collected on P12, P17, P21, P25, and P30 and prepared as retinal whole mounts for further evaluations. From P17, only pups with body weight above 6 g were used for examinations (Stahl et al., 2010a). To overcome biological variations, samples in each time point were randomly selected from different litters. Pups of same age and always held in a room air (RA) environment served as control groups. Six to eight pups were included in each group at various time points. Both males and females were used.

### 2.3 Microglia inhibition by intravitreal injection of liposomes-clodronate

Microglia inhibition was achieved by intravitreal injection of 1 μl of liposomes containing clodronate (CLD, Clodronate-liposomes, Netherlands). In this procedure, the neonatal mouse pup was anesthetized by inhaling a gas mixture with 2% halothane in 70% nitrous oxide and 30% oxygen. The animal was placed on a heating pad to maintain the body temperature at 37°C. The eyelid was opened using a 33½ G needle (Becton, Dickinson and Company, NJ, United States). The cornea was topically anesthetized using 0.5% Alcaine (Alcon, Alcon-Couvreur, Belgium), and the pupil was dilated by topical 1% Mydricyl (Alcon, Alcon-Couvreur, Belgium). The intravitreal injection was performed using a Hamilton syringe through a 34½ needle (Hamilton Company, NV, United States). The needle was inserted into the vitreous cavity through a puncture point in the inferotemporal quadrant immediately below the limbus so as not to damage the lens. One eye of the pup received 1 μl of liposomes-CLD, while the other eye received the same volume of liposomes-PBS (phosphate-buffered saline) as the control. Each pup received one single intravitreal injection in each eye on either P5 before hyperoxic treatment, or on P12 immediately after hyperoxic treatment.

### 2.4 Retinal vasculature morphology

#### 2.4.1 Retinal vasculature and microglia immunohistochemistry in whole-mounted retinae

Whole-mount retinae were fixed by 4% paraformaldehyde/PBS on ice for 1–2 h and then permeabilized in ice-cold ethanol (70%) for 20 min, followed by 1% Triton X-100/PBS (Sigma-Aldrich Corporation, St. Louis, MO) for 30 min at room temperature. After blocking with 10% goat serum in PBS for 1 h at room temperature, the retinal whole mounts were incubated with Alexa Fluor-488-conjugated *Griffonia simplicifolia* isolectin B4 (*GS*-IB4, 0.1 mg/ml; catalog #I21411, Invitrogen Corporation, Carlsbad, CA) for retinal vessel labeling and rabbit anti-ionized calcium-binding adapter molecule 1 (Iba-1, 1:800; catalog # 019–19741, Wako Chemicals United States, Richmond, VA) for microglia staining, respectively, for 72 h incubation at 4°C with gentle shaking. Iba-1 signal was probed using goat anti-rabbit IgG-conjugated with Alexa Fluor-568 (1:500; catalog # A11011, Molecular Probes; Invitrogen Corporation, Carlsbad, CA) for 1 h at room temperature. After three times of PBS washes, retina samples were cover slipped, and stored at −20°C for further use.

#### 2.4.2 Central vaso-obliteration assessment

Retinal whole mounts were stained by Alexa Fluor-488-conjugated GS-IB4 (0.1 mg/ml; Invitrogen, Carlsbad, CA) for retinal vessel visualization. Images of superficial retinal vessels were taken under a fluorescence microscope (Eclipse 80i, Nikon, Japan) using ×40 magnification. The central vascular obliteration area was outlined and measured using Photoshop CS5 (Adobe Systems Inc., San Jose, CA). During the analysis, the outer border of the vascularized retina was first outlined. The borders of the avascular areas were then traced. The central obliteration area and the total retina area in pixels were computed, and the percentage of the central obliterated area over the total retinal area was calculated.

#### 2.4.3 Assessment of retinal NVs by fluorescein angiography

GS-IB4 showed a high affinity with retinal vessels, and thus higher intensity was observed in neovascular tufts. Isolectin-stained whole-mounted retinae were then used for NV assessment as previously published (Fu et al., 2012). Briefly, in retinal whole-mount images, the tuft area was first selected by hand with the Magic Wand tool (Photoshop CS5; Adobe Systems Inc., San Jose, CA). The tolerance value was set to 50, and the “anti-alias” and “contiguous” functions were both enabled during the measurement, so that the NV areas could be sharply selected with no adjacent non-NV areas included. In addition, the total surface area of the retina was outlined along the outer border of the vascularized retinal area. The percentage of NV area in pixels over the total retinal area in pixels was calculated.

### 2.5 Retinal microglia categorization and quantification

Retinal images were collected under a confocal laser scanning microscope using ×200 magnification (LSM700; Carl Zeiss, Thornwood, NY). Each visual field was set at 400 × 400 μm^2^ in size. Superficial and deep retinal vessels were distinguished by adjusting the scanning plane of a confocal microscope. Superficial vessels represented the vascular plexus around the ganglion cell layer (GCL), while deep vessels were the vascular plexus near the outer plexiform layer (OPL). A series of images were scanned using the Z-stack function in each vascular layer and then stacked into one single image for further analyses. The flat-mounted retinae were divided into central and mid-peripheral areas. The central retina was defined as the area within one visual field distance away from the center of a retina under ×200 magnification, while the mid-peripheral retina was defined as the area located halfway from the center to the peripheral border of a retina (Fu et al., 2012). For P17 flat-mounted retinal samples, the mid-peripheral retina was further divided into tuft area and non-tuft area. Six to eight visual fields were randomly selected in each retinal area. Ramified and amoeboid microglia were differentiated according to their morphological characteristics: ramified microglia possessed a small cell body but a few thin and long processes with branches. Amoeboid microglia in contrast had a round shape in morphology, or an enlarged cell body with fewer short and thick processes (Vessey et al., 2011). Microglia in each image were counted three times to obtain an average number, which was further converted into the number of microglia/mm^2^. All the average numbers from different visual fields in a certain retinal area (e.g., central superficial retina) were pooled together and then averaged to obtain the estimated number of microglia in that area of each retina. Ramified and amoeboid microglia were quantified, respectively, in each image. In each image, the numbers of amoeboid and ramified microglia were added to obtain the total number of microglia, termed as total microglia.

### 2.6 Retinal microglia and vessel relationship analyses

In each image of P21, P25, P30, and deep mid-peripheral P17 retinae, the number of vascular meshes formed by retinal vessels and the total length of the retinal vessels were calculated using the angiogenesis plugin of ImageJ software (NIH, Bethesda, MD). The differences between RA and OIR groups were compared. At the same time, the numbers of amoeboid microglia and total microglia were counted in the same image. In RA retinae, as amoeboid microglia seldom exist, only the number of total microglia was counted for later analysis. The numbers of microglia (amoeboid and total microglia) and the calculations of retinal vessels (the number of vascular meshes and total vessel length) of each image were plotted in a Cartesian coordinate system. Linear regression analyses were performed to test the relationship between microglia and retinal vessels.

### 2.7 Study design and statistical analysis

All experiments and analyses were performed in a blinded manner. The unpaired *t-*test, *one-way analysis of variance (ANOVA)*, and *Bonferroni’s* multiple comparison tests were used for comparison of results as specified in the figure legends (Prism v5.0, GraphPad Software Inc., San Diego, CA, United States). Statistical significance was set at *p* < 0.05. Data were presented as mean ± SEM.

## 3 Result

### 3.1 Retinal vessel changes during OIR

On P12, room air (RA) group retinae showed normal retinal vasculature development (Figure 1B). The whole retina was covered with retinal vessels, with no avascular zone or NVs observed. The retinal vasculature displayed normal organization in RA retinae on P17, P21, P25, and P30 (Figures 1C,G–I). On P12, immediately after hyperoxia treatment, an avascular zone was observed in the central part of the OIR retina (Figure 1D). Hyperoxia led to a halt of normal retinal vessel development and regression of existing retinal vessels in both superficial and deep retinal vascular plexuses. From P12 onwards, the room air environment resulted in a relative hypoxic stress to OIR retinae, leading to occurrence of NVs in the mid-peripheral retina, which peaked on P17 (Figure 1E). The avascular zone in OIR retinae regressed gradually (Figures 1D–F), and the NVs began to regress after P17. By P21 and afterward, the OIR retinae were fully covered with blood vessels, with no obvious NVs observed (Figures 1J–L).

**FIGURE 2:**
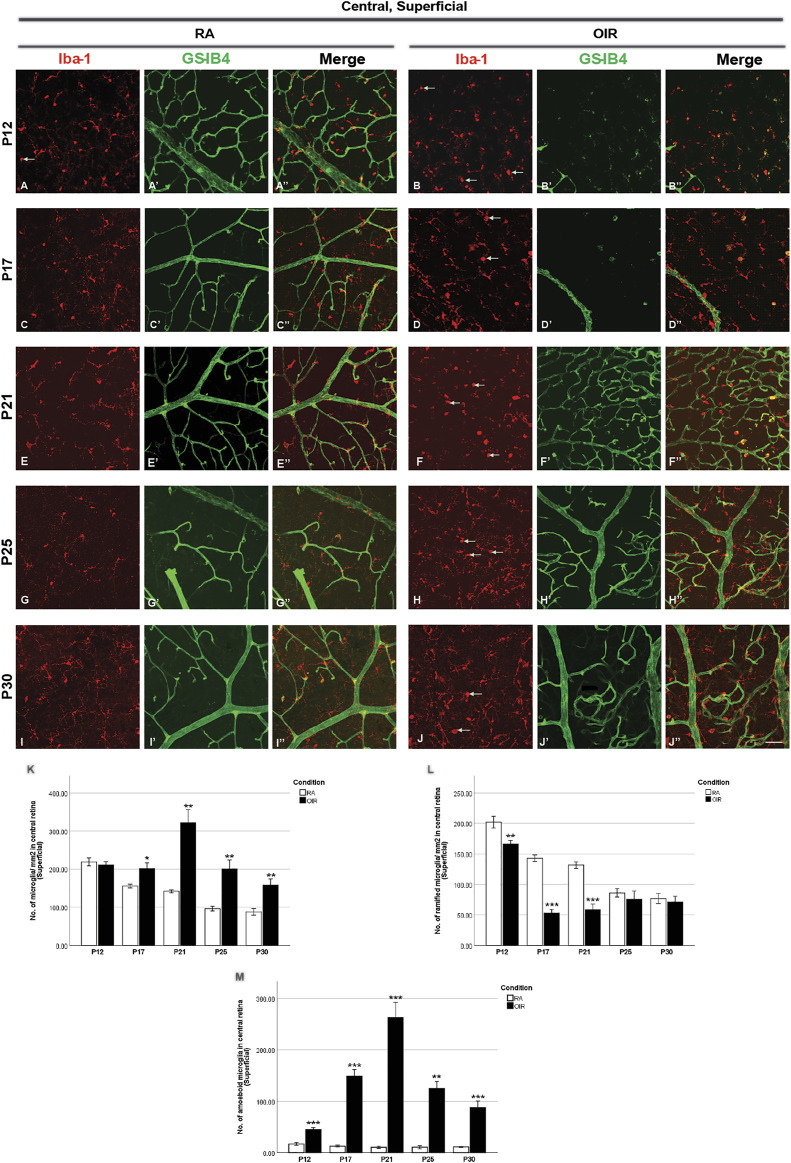
Representative images of the superficial central retinal area stained for microglia and retinal vessels from P12 to P30 in whole-mounted retinae. Microglia were stained with Iba-1 (red, **(A–J)**), and the blood vessels were stained with GS-IB4 (green, **(A’–J’)**). The association between microglia and blood vessels can be indicated in the merged images **(A’’–J’’)**. Ramified microglia dominated the microglia population, while occasionally amoeboid microglia (indicated by arrows) could be observed in RA retinae **(A)**. Hyperoxia treatment led to blood vessel regression in the central part of OIR retinae on P12 and P17 **(B’,D’)**. More retinal vessels were observed in OIR superficial retinae after P17 **(F’,H’,J’)**. The number of total, ramified, and amoeboid microglia was quantified **(K–M)**. In central OIR retinae, decreased ramified microglia from P12 to P21 and increased amoeboid microglia from P12 to P30 were observed **(L,M)**. In addition, increased total microglia was also observed in central OIR retinae from P17 to P30 **(K)**. *n* = 6–8 in each group. *p < 0.05, **p < 0.01, ***p < 0.001, compared with RA, unpaired *t*-test. Scale bar, 50 μm.

**FIGURE 3:**
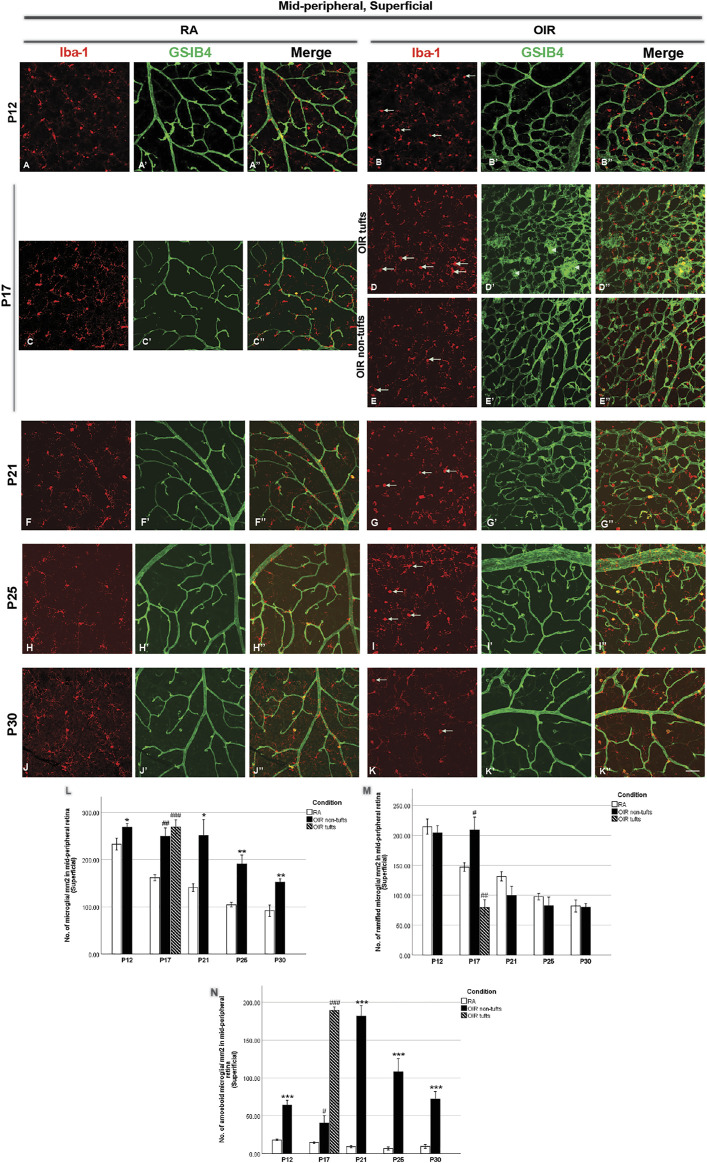
Representative images of the superficial mid-peripheral retinal area stained for microglia and retinal vessels from P12 to P30 in whole-mounted retinae. Microglia were stained with Iba-1 (red, **(A–K)**), and the blood vessels were stained with GS-IB4 (green, **(A’–K’)**). The association between microglia and blood vessels can be indicated in the merged images **(A’’–K’’)**. In mid-peripheral retinae, increased amoeboid and total microglia were observed in OIR retinae on P12 **(A,B,M,N)**. On P17, NV tufts could be observed (arrowheads, **(D’)**) in OIR retinae. In tuft areas of OIR retinae, increased amoeboid and total, but decreased ramified microglia were observed **(D,D’’,L–N)**; in non-tuft retinal areas of OIR retina, increases in the numbers of total and ramified microglia were observed, but decreased amoeboid microglia were observed **(E,E’’,L–N)**. Moreover, increased amoeboid and total microglia were observed in OIR retinae from P21 to P30 **(L,N)**. n = 6–8 in each group. *p < 0.05, **p < 0.01, ***p < 0.001, compared with RA, unpaired *t*-test or ^#^ *p* < 0.05, ^##^ *p* < 0.01, ^###^ *p* < 0.001, compared with RA*, one-way ANOVA* followed by *Bonferroni’s* test. Scale bar, 50 μm.

**FIGURE 4:**
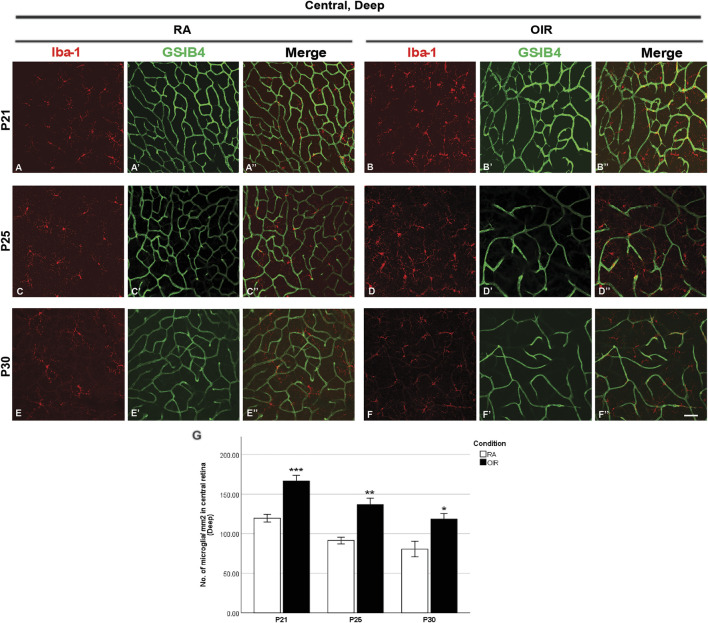
Representative images of the deep central retinal area stained for microglia and retinal vessels from P21 to P30 in whole-mounted retinae. Microglia were stained with Iba-1 (red, **(A–F)**), and the blood vessels were stained with GS-IB4 (green, **(A’–F’)**). The association between microglia and blood vessels can be indicated in the merged images **(A’’–F’’)**. Increased total microglia were observed in central OIR retinae compared with RA from P21 to P30 in the deep central retinal area **(A–G)**. n = 6–8 in each group. *p < 0.05, **p < 0.01, ***p < 0.001, compared with RA, unpaired *t*-test. Scale bar, 50 μm.

**FIGURE 5:**
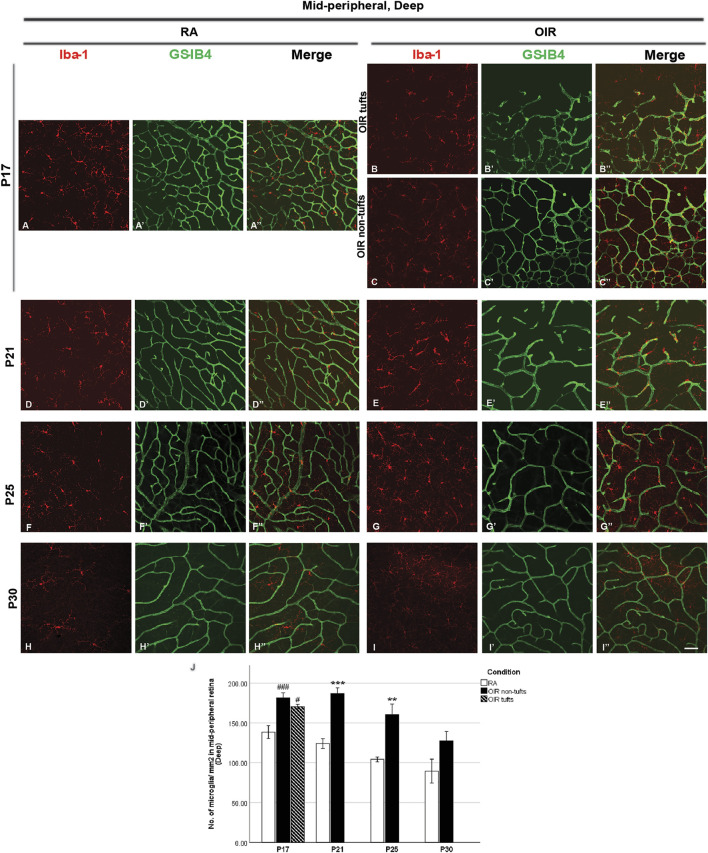
Representative images of deep mid-peripheral retinal area stained for microglia and retinal vessels from P17 to P30 in whole-mounted retinae. Microglia were stained with Iba-1 (red,** A–I**), and the blood vessels were stained with GS-IB4 (green, **A’–I’**). The association between microglia and blood vessels can be indicated in the merged images **(** **A’’–I’’** **)**. Increased total microglia were observed in midperipheral tuft and non-tuft retinae on P17 and OIR retinae compared with RA ones from P21 to P25 in the deep mid-peripheral retinal area, whereas this increase was mild on P30OIR retinae **(A–J)**. n = 6–8 in each group. **p < 0.01, ***p < 0.001, compared with RA, unpaired *t*-test or ^#^ *p* < 0.05, ^###^ *p* < 0.001, compared with RA*, one-way ANOVA* followed by *Bonferroni’s* test. Scale bar, 50 μm.

**FIGURE 6:**
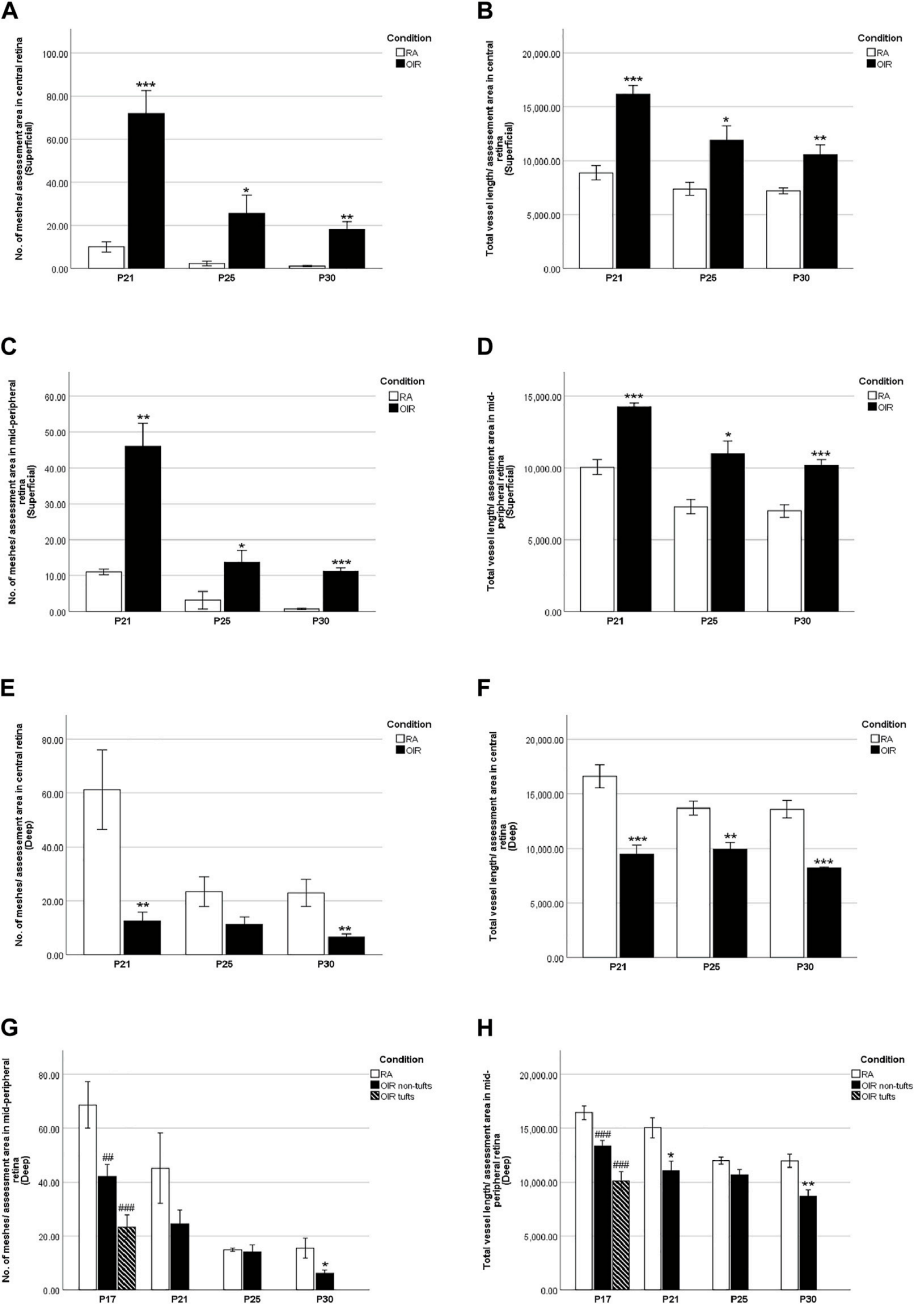
Retinal vascular meshes and total vessel length in RA and OIR retinae. The number of retinal vascular meshes **(A,C,E,G)** and the total length of retinal vessels **(B,D,F,H)** were calculated and compared between RA and OIR. More retinal vessels with increased vascular meshes and total vessel length were observed in superficial central and mid-peripheral retinal areas in OIR retinae on P21, P25, and P30 compared with RA **(A,B,C,D)**. For the deep central retinal area, decreased vascular meshes on P21 and P30 and decreased total vessel length from P21 to P30 were observed compared with RA **(E,F)**. On P17, decreased vascular meshes and total vessel length were observed in both tuft and non-tuft areas in deep mid-peripheral OIR retinae compared with RA **(G,H)**. In addition, fewer retinal vessels were observed on P21 with decreased total vessel length and on P30 with decreased vascular meshes and vessel length compared with RA **(G,H)**. (n = 6–8 in each group. *p < 0.05, **p < 0.01, ***p < 0.001, compared with RA, unpaired *t*-test or ^##^ *p* < 0.01, ^###^ *p* < 0.001, compared with RA*, one-way ANOVA* followed by *Bonferroni’s* test).

We further examined the superficial and deep layer vascular patterns in the central and mid-peripheral retina (Figures 2–5). Overall, there was an increased number of meshes and total vessel length per field in the superficial vascular plexus in OIR vs. RA retinae (Figures 6A–D), suggesting delayed vascular pruning. Moreover, there is decreased vascular density in the deep vascular plexus (Figures 6E–H), suggesting delayed vascularization.

At P17, deep OIR mid-peripheral retinae showed fewer vascular meshes [RA: 68.7 ± 6.3, OIR tufts: 23.2 ± 4.6 (*p* < 0.001), and OIR non-tufts: 42.1 ± 4.4 (*p* < 0.01)/assessment area] and less total vessel length [RA: 16442 ± 509.3, OIR tufts: 10118 ± 865.0 (*p* < 0.001), and OIR non-tufts: 13359 ± 483.8 (*p* < 0.001)/assessment area] (Figures 5A’–C’, Figures 6G,H).

In P21 OIR retinae, the number of vascular meshes formed in each assessment area in the central superficial retina was increased compared with RA retinae (RA: 10.0 ± 2.4 vs. OIR: 72.0 ± 10.5/assessment area, *p* < 0.001); similar increase was also observed for the total length of retinal vessels in each assessment area (RA: 8883 ± 670.5 vs. OIR: 16150 ± 844.6/assessment area, *p* < 0.001) (Figures 2E’,F’, Figures 6A,B). More retinal vessels were observed in mid-peripheral superficial OIR retinae. OIR retinae displayed more vascular meshes (RA: 13.3 ± 2.3 vs. OIR: 45.8 ± 5.2/assessment area, *p* < 0.01) and increased total length (RA: 10050 ± 522.2 vs. 14250 ± 277.5/assessment area, *p* < 0.001) compared with RA retinae (Figures 3F’,G’, Figures 6C,D). However, less vascular meshes were formed in the same areas of OIR central deep retinae than RA ones (RA: 61.2 ± 14.8 vs. OIR: 12.4 ± 3.5/assessment area, *p* < 0.01), as well as total retinal vessel length (RA: 16,620 ± 1,050 vs. OIR: 9,483 ± 806.8/assessment area, *p* < 0.001) (Figures 4A’,B’, Figures 6E,F). In the mid-peripheral deep retina, OIR retinae showed significantly decreased total vessel length compared with RA ones (RA: 15040 ± 951.4 vs. OIR: 11060 ± 883.9/assessment area, *p* < 0.05), while the decrease in the number of vascular meshes was not significant (RA: 45.18 ± 13.1 vs. OIR: 24.5 ± 5.1/assessment area, *p* = 0.1503) (Figures 5D’,E’, Figures 6G,H).

P25 OIR retinae showed increased number of vascular meshes (RA: 2.4 ± 1.1 vs. 25.6 ± 8.3/assessment area, *p* < 0.05) and more total vessel length (RA: 7373 ± 602.6 vs. OIR: 11900 ± 1348/assessment area, *p* < 0.05) (Figures 2G’,H’, Figures 6A,B) in central superficial retinae. More retinal vessels were observed in OIR retinae than RA retinae, with more vascular meshes (RA: 3.2 ± 2.5 vs. OIR: 13.7 ± 3.3/assessment area, *p* < 0.05) and increased total vessel length (RA: 7,291 ± 499.1 vs. OIR: 11000 ± 877.1/assessment area, *p* < 0.05) (Figures 3H’,I’, Figures 6C,D) in mid-peripheral superficial retinae. However, decreased retinal vessels were observed in central deep OIR retinae (vascular meshes: RA: 23.4 ± 5.6 vs. OIR: 11.3 ± 2.7/assessment area, *p* = 0.0604; total vessel length: RA: 13,700 ± 641.8 vs. OIR: 9,956 ± 595.1/assessment area, *p* < 0.01; Figures 4C’,D’, Figures 6E,F). The amounts of retinal vessels at mid-peripheral deep retinal area are comparable between RA and OIR groups (vascular meshes: RA: 14.9 ± 0.6 vs. OIR: 14.1 ± 2.7/assessment area, *p* = 0.8034; total vessel length: RA: 12000 ± 346.9 vs. OIR: 10670 ± 515.5/assessment area, *p* = 0.0951; Figures 5F’,G’, Figures 6G,H).

P30 OIR retinae showed more vascular meshes (RA: 1.1 ± 0.4 vs. OIR: 18.13 ± 3.6/assessment area, *p* < 0.01) and increased total vessel length (RA: 7,201 ± 284.5 vs. OIR: 10550 ± 900.9, *p* < 0.01) (Figures 2I’,J’, Figures 6A,B) in central superficial retinal areas. OIR retinae also showed increased vascular meshes (RA: 0.8 ± 0.2 vs. OIR: 11.2 ± 1.1/assessment area, *p* < 0.001) and total vessel length (RA: 7,005 ± 445.4 vs. OIR: 10190 ± 369.6/assessment area, *p* < 0.001) (Figures 3J’,K’, Figures 6C,D) in the mid-peripheral superficial retina. In the central deep retina, OIR retinae showed decreased number in vascular meshes (RA: 22.9 ± 5.0 vs. OIR: 6.6 ± 1.1/assessment area, *p* < 0.01) and reduced total vessel length (RA: 13610 ± 818.1 vs. OIR: 8,216 ± 72.6/assessment area, *p* < 0.001) compared with RA retinae (Figures 4E’,F’, Figures 6E,F). In the mid-peripheral deep retina, OIR retinae formed less vascular meshes (RA: 15.5 ± 3.7 vs. OIR: 6.2 ± 1.3/assessment area, *p* < 0.05) and displayed reduced total vessel length (RA: 11970 ± 597.0 vs. OIR: 8,702 ± 586.3/assessment area, *p* < 0.01) (Figures 5H’,I’, Figures 6G,H).

Taken together, these findings suggest that there was persistent vascular damage even after the VO and NV were resolved in OIR.

### 3.2 Retinal microglial changes during OIR

We next analyzed retinal microglial status at P12, P17, P21, P25, and P30 (Figures 2–5). In general, microglia were mostly present in the ramified form under RA conditions, and there was a substantial reduction from P12 to P30 during the physiological development (Figure 2L, Figure 3M). In OIR, there was a further decrease in ramified microglia from P12 to P21 (Figure 2L, Figure 3M). Meanwhile, there was a profound increase in amoeboid microglia in the superficial vascular layer at the central and mid-peripheral areas starting from P12. The number peaked at P21 and decreased afterward (Figure 2M, Figure 3N). Furthermore, the number of ramified microglia was increased in the deep vascular layer in OIR vs. RA groups after P12 (Figure 4G, Figure 5J).

#### 3.2.1 Analysis of retinal microglia on P12

Five-day hyperoxia treatment led to retinal vasculature ablation in the central retina on P12 (Figure 1D, Figure 2B’), stopping the development of normal retinal vessels. Thus, no retinal capillaries could be observed in either the central superficial OIR retina or central deep OIR retina (Fu et al., 2012). In RA retinae, the majority of the microglia population displayed a ramified morphology, with a very small number of amoeboid microglia observed occasionally. In the central superficial retina, OIR retinae showed an increase in amoeboid microglia (RA: 16.9 ± 2.5 vs. OIR: 44.8 ± 3.9/mm^2^, *p* < 0.0001) but a decrease in ramified microglia (RA: 202.1 ± 9.6 vs. OIR: 165.9 ± 6.0/mm^2^, *p* < 0.01) when compared with RA retinae. The numbers of total microglia were comparable in both groups (RA: 218.9 ± 10.4 vs. OIR: 210.7 ± 9.0, *p* = 0.5648) (Figures 2A,B,K–M). In the mid-peripheral superficial retina, increased amoeboid microglia (RA: 18.1 ± 0.9 vs. OIR: 64.2 ± 6.2/mm^2^, *p* < 0.0001) and total microglia (RA: 232.6 ± 12.5 vs. OIR: 268.4 ± 8.3/mm^2^, *p* < 0.05) were observed in OIR retinae compared with RA retinae (Figures 3A,B,L–N); RA and OIR retinae showed comparable amounts of ramified microglia (RA: 214.5 ± 12.8 vs. OIR: 204.2 ± 12.0/mm^2^, *p* = 0.5686). No obvious differences were observed in the number of deep microglia between RA and OIR retinae (Central: RA: 220.4 ± 13.5 vs. OIR: 249.2 ± 17.7, *p* = 0.2115; mid-peripheral: RA: 212.5 ± 11.4 vs. OIR: 238.6 ± 17.7, *p* = 0.2257).

#### 3.2.2 Analysis of retinal microglia and vessels on P17

NVs peaked on P17, with NV tufts observed in the mid-peripheral superficial retina (Figure 1E, Figure 3D’). Although the central avascular zone regressed, a smaller avascular zone could still be observed in OIR retinae on P17 (Figure 1E, Figure 2D’) (Fu et al., 2012).

In the central superficial retina, increased total microglia were observed in OIR retinae compared with RA retinae (RA: 155.6 ± 4.7 vs. OIR: 201.1 ± 15.1/mm^2^, *p* < 0.05), which was mainly due to the increase in number of activated amoeboid microglia (RA: 12.8 ± 1.9 vs. OIR: 148.3 ± 13.4/mm^2^, *p* < 0.001); however, the number of ramified microglia was decreased in the OIR retina (RA: 142.8 ± 5.3 vs. 52.8 ± 6.4/mm^2^, *p* < 0.001) (Figures 2C,D,K–M). No obvious differences in the number of ramified microglia between RA and OIR retinae were observed in the central deep retina (RA: 130.9 ± 6.5 vs. 147.6 ± 14.7/mm^2^, *p* = 0.3253). These findings suggested the transformation of microglia from ramified into amoeboid form and microglial activation in the central superficial retina.

In the mid-peripheral superficial retina, the retinal area was further categorized into tuft area and non-tuft area. In tuft area, an increased number in total (RA: 161.6 ± 6.6 vs. OIR: 269.2 ± 15.2/mm^2^, *p* < 0.001) and amoeboid (RA: 14.6 ± 1.0 vs. OIR: 189.5 ± 4.5/mm^2^, *p* < 0.001) microglia but a decreased number in ramified (RA: 147.0 ± 7.1 vs. OIR: 79.7 ± 13.0/mm^2^, *p* < 0.01) microglia was observed in OIR retinae when compared with RA retinae (Figures 3C, D,L–N). In the non-tuft area, the total number of microglia (OIR: 249.1 ± 17.8/mm^2^) was increased in OIR retinae (*p* < 0.01), with an increase in both amoeboid (OIR: 40.1 ± 10.0/mm^2^, *p* < 0.05) and ramified (OIR: 209.0 ± 21.5/mm^2^, *p* < 0.05) microglia when compared with RA retinae (Figures 3C,E,L–N). In mid-peripheral deep retinae, increased ramified microglia were observed in OIR retinae compared with RA retinae (RA: 138.4 ± 7.9, OIR tufts: 170.4 ± 2.9, OIR non-tufts: 181.4 ± 6.4/mm^2^) (Figures 5A–C,J). These observations suggest potential microglial proliferation with the increased total number.

We then examined the association between the mid-peripheral deep vascular pattern and microglial status. No significant association was found between the number of vascular meshes and the number of total microglia under RA and OIR conditions (RA: *r* ^2^ = 0.0411, *p* = 0.3659, Figure 10A; OIR tufts: *r* ^2^ = 0.1364, *p* = 0.3280, Figure 10B; OIR non-tufts: *r* ^2^ = 0.2072, *p* = 0.0882, Figure 10C). For the relationship between the total vessel length and the number of total microglia, still no significant correlation was found (RA: *r* ^2^ = 0.0645, *p* < 0.2542, Figure 10A’; OIR tufts: *r* ^2^ = 0.1455, *p* = 0.3122, Figure 10B’; OIR non-tufts: *r* ^2^ = 0.2207, *p* = 0.0773, Figure 10C’).

#### 3.2.3 Analysis of retinal microglia and vessels on P21

**FIGURE 7:**
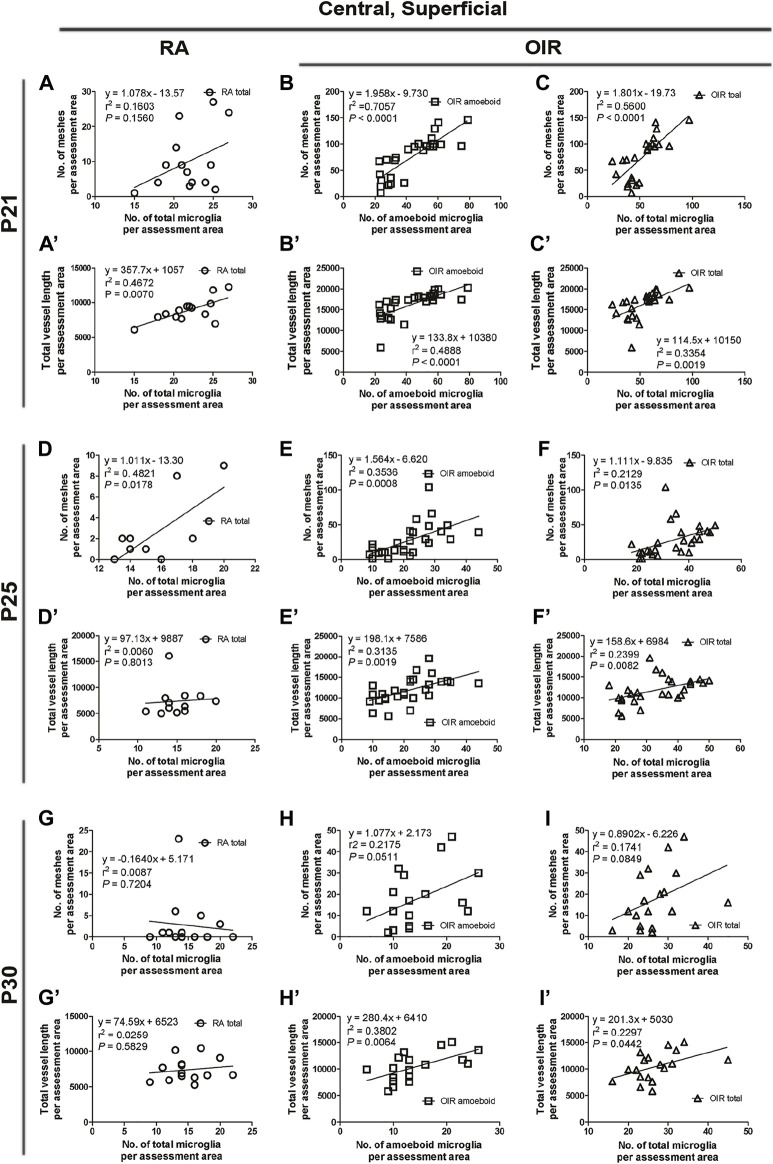
Correlation between microglia and retinal vessels of the superficial central retinal area from P21 to P30 under RA and after OIR. The association between microglia (amoeboid and total microglia) and retinal vessels (number of vascular meshes and total vessel length) in the superficial central retina was analyzed by linear regression. In RA retinae, the total vessel length was associated with the number of total microglia on P21 **(A)** and the number of vascular meshes was found to be associated with the number of total microglia on P25 **(D)**, whereas such association was not observed between the number of vascular meshes and the number of total microglia on P21 **(A’)** or between total vessel length and the number of total microglia on P25 **(D’)**. On P30, the association between retinal vessels and microglia was not observed **(G,G’)**. In P21 OIR retinae, the number of vascular meshes and the total length of retinal vessels were positively associated with the number of amoeboid microglia and total microglia **(B,B’,C,C’)**. On P25, the number of vascular meshes and the total length of retinal vessels were positively associated with the number of amoeboid microglia and total microglia **(E,E’)**. This association was also observed with the number of total microglia in OIR retinae **(F,F’)**. In P30 OIR retinae, the total length of retinal vessels was positively associated with the number of amoeboid microglia **(H’)** and with the number of total microglia **(I’)**; however, this association was not found between the number of vascular meshes and the number of retinal microglia **(H,I)**.

On P21, the avascular zone and the NVs could be barely observed in OIR retinae. In the central superficial retina, there were increased total (RA: 141.9 ± 3.6 vs. OIR: 321.8 ± 34.2/mm^2^, *p* < 0.01) and amoeboid microglia (RA: 10.4 ± 2.2 vs. OIR: 263.2 ± 29.1/mm^2^, *p* < 0.001) but decreased ramified (RA: 131.5 ± 5.4 vs. 58.6 ± 9.0/mm^2^, *p* < 0.001) microglia in OIR retinae when compared with RA retinae (Figures 2E, F,K–M). In RA retinae, the total vessel length was associated with the number of total microglia (*r* ^2^ = 0.4672, *p* = 0.0070, Figure 2E”, Figure 7A’), while the association between the number of vascular meshes and the number of total microglia was not significant (*r* ^2^ = 0.1603, *p* = 0.1560, Figure 2E”, Figure 7A). In OIR retinae, the number of vascular meshes and the total length of retinal vessels were positively associated with the number of amoeboid microglia (vascular meshes: *r* ^2^ = 0.7057, *p* < 0.0001, Figure 2F”, Figure 7B; vessel length: *r* ^2^ = 0.4888, *p* < 0.0001, Figure 2F”, Figure 7B’). In OIR retinae, the number of vascular meshes and the total length of vessels were also associated with the total number of microglia (vascular meshes: *r* ^2^ = 0.5600, *p* < 0.0001, Figure 2F”, Figure 7C; vessel length: *r* ^2^ = 0.3354, *p* = 0.0019, Figure 2F”, Figure 7C’).

**FIGURE 8:**
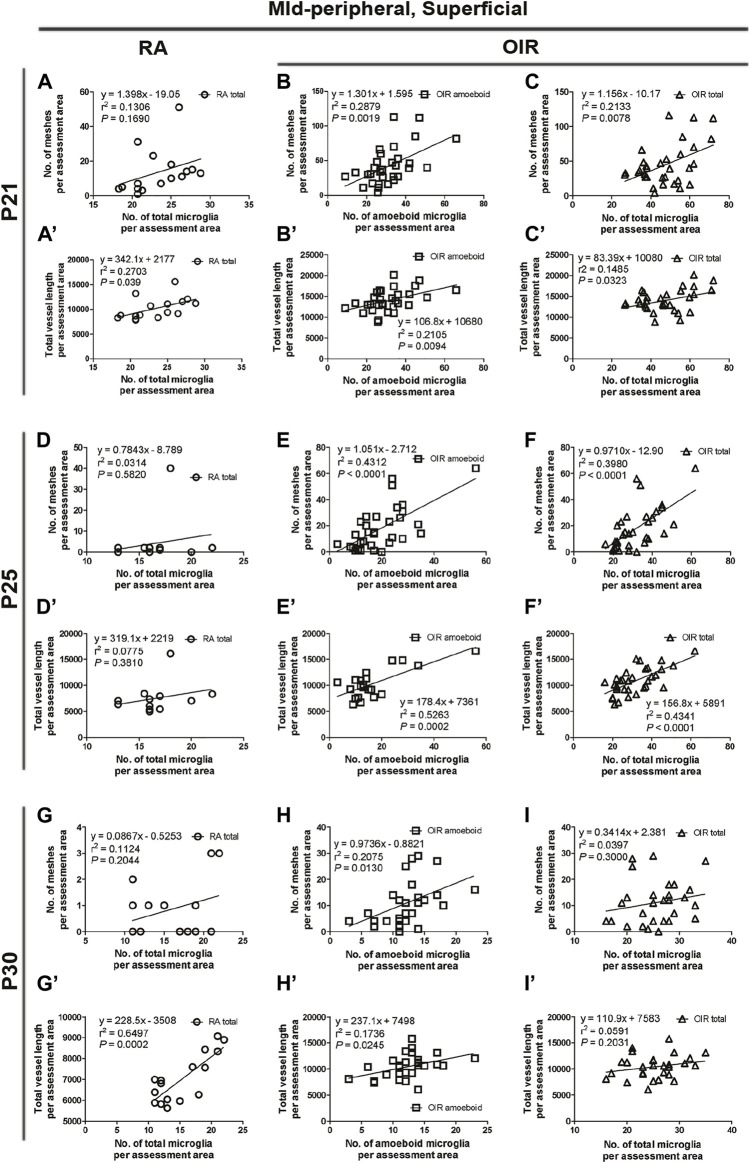
Correlation between microglia and retinal vessels of the superficial mid-peripheral retinal area from P21 to P30 under RA and after OIR. The association between microglia (amoeboid and total microglia) and retinal vessels (number of vascular meshes and total vessel length) in the superficial mid-peripheral retina was analyzed by linear regression. In RA retinae, the total vessel length was associated with the number of total microglia **(A’)**, while the association was not significant between the number of vascular meshes and total microglia number **(A)** on P21. However, no association was observed between the retinal vessels and the number of total microglia on P25 **(D,D’)**. In P30 RA retinae, the total vessel length, but not the number of vascular meshes, was positively associated with the number of total microglia **(G,G’)**. After OIR, the number of amoeboid microglia and the total number of microglia were positively associated with the number of vascular meshes **(B,C,E,F)**, as well as the total length of retinal vessels **(B’,C’,E’,F’)** on P21 and P25. On P30, the retinal vessels were positively associated with the number of amoeboid microglia **(H,H’)**; however, the associations were not found with the number of total microglia in OIR retinae **(I,I’)**.

In the mid-peripheral superficial retina, OIR retinae showed increased total microglia (OIR: 251.0 ± 33.6/mm^2^) with a dominant increase in amoeboid form (OIR: 151.6 ± 32.4/mm^2^) when compared with RA retinae (RA total: 140.6 ± 8.3/mm^2^, *p* < 0.05; RA amoeboid: 9.1 ± 1.6/mm^2^, *p* < 0.01), while the number of ramified microglia was comparable (RA: 131.5 ± 7.7 vs. OIR: 99.5 ± 15.4/mm^2^, *p* = 0.1165) (Figures 3F, G,L–N). In RA retinae, the total vessel length was associated with the number of total microglia (*r* ^2^ = 0.2703, *p* = 0.039, Figure 3F”, Figure 8A’), while the association was not significant between the number of vascular meshes and total microglia number (*r* ^2^ = 0.1306, *p* = 0.1690, Figure 3F”, Figure 8A). In OIR retinae, the number of amoeboid microglia was positively associated with the number of vascular meshes (*r* ^2^ = 0.2879, *p* = 0.0019, Figure 3G”, Figure 8B), as well as total length of retinal vessels (*r* ^2^ = 0.2105, *p* = 0.0094, Figure 3G”, Figure 8B’). Similar association was also found between the total number of microglia and retinal vessels (vascular meshes: *r* ^2^ = 0.2133, *p* = 0.0078; total length: *r* ^2^ = 0.1485, *p* = 0.0323) in OIR retinae (Figure 3G”, Figures 8C,C’).

**FIGURE 9:**
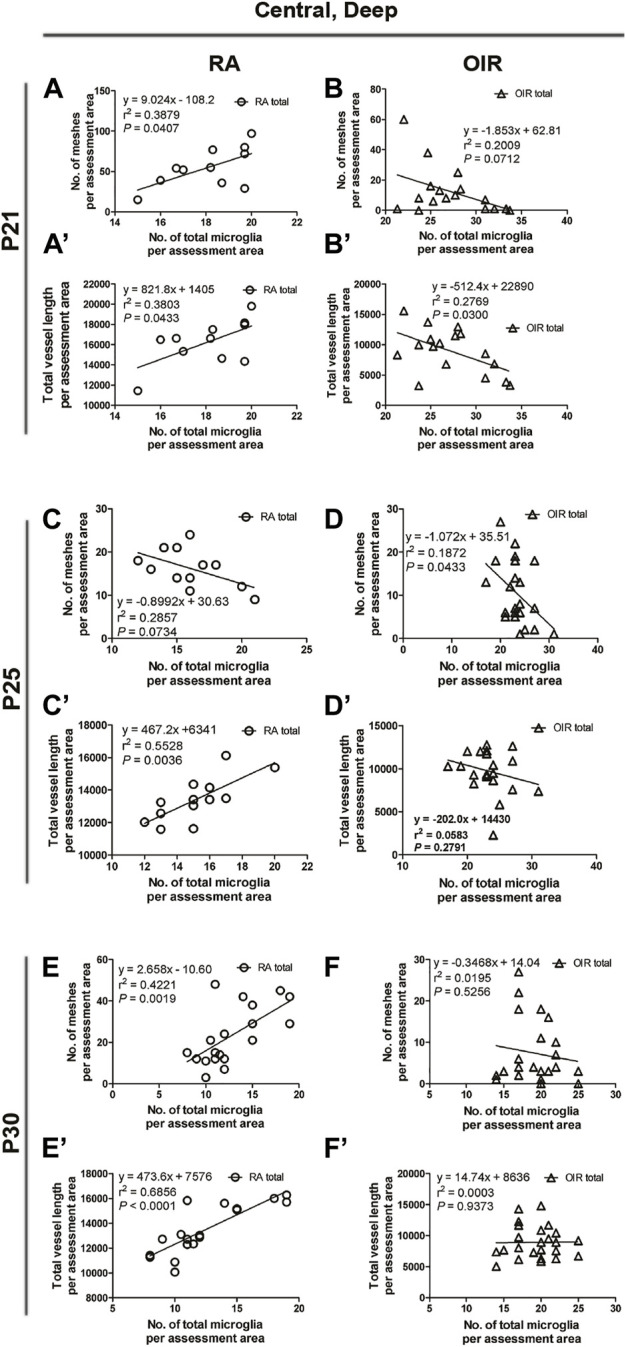
Correlation between microglia and retinal vessels of the deep central retinal area from P21 to P30 under RA and after OIR. The association between microglia and retinal vessels (number of vascular meshes and total vessel length) in the deep central retina was analyzed by linear regression. In RA retinae, the number of vascular meshes and the total vessel length were positively associated with the number of microglia on P21 **(A,A’)** and P30 **(E,E’)**. In P25 RA retinae, the total vessel length was positively associated with the total number of microglia **(C’)**, whereas this association was not found between the number of vascular meshes and the number of total microglia **(C)**. In OIR retinae, a negative association was observed between the number of vascular meshes and the number of total microglia on P21 **(B,B’)** and between the total length of deep retinal vessels and the total number of microglia on P25 **(D,D’)**. However, no association was observed between the number of retinal vessels and retinal microglia number on P30 **(F,F’)**.

**FIGURE 10:**
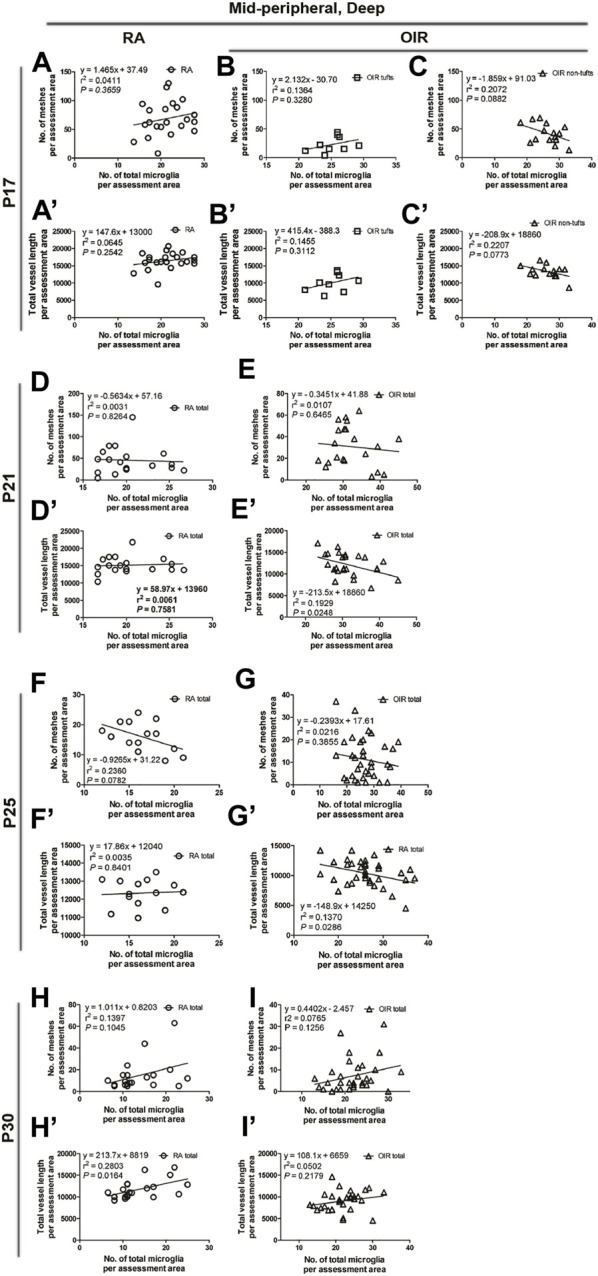
Correlation between microglia and retinal vessels of the deep mid-peripheral retinal area from P17 to P30 under RA and after OIR. The association between microglia and retinal vessels (number of vascular meshes and total vessel length) in the deep mid-peripheral retina was analyzed by linear regression. In RA retinae, no significant association was found between the number of vascular meshes and the number of total microglia and between total vessel length and the number of total microglia on P17, P21, and P25 **(A,A’,D,D’,F,F’)**, whereas a positive association was observed between the total vessel length and the number of total microglia on P30 **(H,H’)**. After OIR, no association was observed between the number of vascular meshes and the number of total microglia and between total vessel length and the number of total microglia on P17 in both tuft and non-tuft areas **(B,B’,C,C’)**. Interestingly, increased microglia were associated with the decreased total length of deep retinal vessels in OIR retinae on P21 and P25 **(E,E’,G,G’)**. On P30, no significant association was found between the retinal vessels and retinal microglia in OIR retinae **(I,I’)**.

In the central deep retina, OIR retinae showed increased ramified microglia when compared with RA retinae (RA: 119.5 ± 4.8 vs. OIR: 166.3 ± 7.4/mm^2^, *p* < 0.001; Figures 4A,B,G); in the mid-peripheral deep retina, increased microglia were observed in OIR retinae (RA: 124.1 ± 6.2 vs. OIR: 186.8 ± 7.3/mm^2^, *p* < 0.001; Figures 5D,E,J). In the central deep areas of RA retinae, the number of vascular meshes and the total vessel length were positively associated with the number of microglia (meshes: *r* ^2^ = 0.3879, *p* = 0.0407; length: *r* ^2^ = 0.3803, *p* = 0.0433; Figure 4A’’, Figures 9A,A’); in the mid-peripheral deep regions of RA retinae, the association was not significant (meshes: *r* ^2^ = 0.0031, *p* = 0.8264; length: *r* ^2^ = 0.0061, *p* = 0.7581; Figure 5D’’, Figures 10D, D’). Interestingly, increased microglia were associated with decreased total length of deep retinal vessels in OIR retinae (central: *r* ^2^ = 0.2769, *p* = 0.0300, Figure 4B’’, Figures 9B’; mid-peripheral: *r* ^2^ = 0.1929, *p* = 0.0248, Figure 5E’’, Figure 10E’); for vascular meshes, their association with microglia was not significant (central: *r* ^2^ = 0.2009, *p* = 0.0712, Figure 4B”, Figure 9B; mid-peripheral: *r* ^2^ = 0.0107, *p* = 0.6465, Figure 5E’’, Figure 10E).

Taken together, at P21, there was a strong association between superficial retinal vasculature and microglial status (total and amoeboid) in OIR. The positive association between central deep retinal vessels and microglia in RA was lost in OIR.

#### 3.2.4 Analysis of retinal microglia and vessels on P25

On P25, in central superficial retinae, there were more total microglia with dominant increase in amoeboid microglia in OIR retinae when compared with RA ones (total microglia: RA: 96.5 ± 6.3 vs. OIR: 200.6 ± 23.1/mm^2^, *p* < 0.01; amoeboid microglia: RA: 10.5 ± 3.0 vs. OIR: 125.0 ± 13.6/mm^2^, *p* < 0.01; Figures 2G, H,K–M). In RA retinae, the number of vascular meshes was found to be associated with the number of total microglia (*r* ^2^ = 0.4821, *p* = 0.0178), whereas such association was not observed between total vessel length and the number of total microglia (*r* ^2^ = 0.0060, *p* = 0.8013) (Figure 2G”, Figures 7D,D’). In OIR retinae, the number of vascular meshes and total retinal vessel length were positively associated with the number of amoeboid microglia (vascular meshes: *r* ^2^ = 0.3536, *p* = 0.0008; total vessel length: *r* ^2^ = 0.3135, *p* = 0.0019; Figure 2H”, Figures 7E,E’). Such associations were also observed with the number of total microglia (vascular meshes: *r* ^2^ = 0.2129, *p* = 0.0135; total vessel length: *r* ^2^ = 0.2399, *p* = 0.0082) in OIR retinae (Figure 2H”, Figures 7F,F’).

In mid-peripheral superficial retinae, increased total (RA: 104.5 ± 5.0 vs. OIR: 190.8 ± 18.9/mm^2^, *p* < 0.01) with increased amoeboid (RA: 6.8 ± 2.1 vs. OIR: 108.1 ± 17.4/mm^2^, *p* < 0.001) microglia were observed in OIR retinae (Figures 3H,I,L–N). In RA retinae, the associations between the retinal vessels and the number of total microglia were not observed (vascular meshes: *r* ^2^ = 0.0314, *p* = 0.5820; total vessel length: *r* ^2^ = 0.0775, *p* = 0.3810) (Figure 3H”, Figures 8D,D’). In OIR retinae, the number of vascular meshes and the total length of retinal vessels were positively associated with the number of amoeboid microglia (vascular meshes: *r* ^2^ = 0.4312, *p* < 0.0001; total vessel length: *r* ^2^ = 0.5263, *p* = 0.0002) (Figure 3I”, Figures 8E,E’). Such associations were also observed with the number of total microglia (vascular meshes: *r* ^2^ = 0.3980, *p* < 0.0001; total vessel length: *r* ^2^ = 0.4341, *p* < 0.0001) in OIR retinae (Figure 3I”, Figures 8F,F’).

In central deep retinae, increased number of ramified microglia was observed in OIR retinae (RA: 91.3 ± 4.2 vs. OIR: 136.7 ± 8.0/mm^2^, *p* < 0.01; Figures 4C,D,G). In RA retinae, the total vessel length was positively associated with the total number of microglia (*r* ^2^ = 0.5528, *p* = 0.0036), whereas this association was not found between the number of vascular meshes and the number of total microglia (*r* ^2^ = 0.2857, *p* = 0.0734) (Figure 4C”, Figures 9C,C’). In OIR retinae, the number of vascular meshes was found negatively associated with the number of total microglia (*r* ^2^ = 0.1872, *p* = 0.0443), while the decrease in total vessel length was not associated with the increase of total microglia (*r* ^2^ = 0.0583, *p* = 0.2791) (Figure 4D”, Figures 9D,D’).

In mid-peripheral deep retinae, increased ramified microglia were also observed in OIR group (RA: 104.3 ± 3.0 vs. OIR: 160.2 ± 13.6/mm^2^, *p* < 0.01; Figures 5F,G,J). In RA retinae, no significant association was found between retinal vessels and microglia (vascular meshes: *r* ^2^ = 0.2360, *p* = 0.0782; total vessel length: *r* ^2^ = 0.0035, *p* = 0.8401; Figure 5F”, Figures 10F,F’). In OIR retinae, the total vessel length was negatively associated with the number of microglia (*r* ^2^ = 0.1370, *p* = 0.0286) in deep mid-peripheral retinae, whereas the association between vascular meshes and microglia was not observed (*r* ^2^ = 0.0216, *p* = 0.3855) (Figure 5G”, Figures 10G,G’).

Taken together, at P25, the strong association between superficial retinal vasculature and microglial status (total and amoeboid) in OIR was preserved. The positive association between central deep retinal vessels and microglia in RA was lost in OIR.

#### 3.2.5 Analysis of retinal microglia and vessels on P30

At P30 in central superficial retinal areas, increased total (RA: 87.8 ± 9.0 vs. OIR: 158.2 ± 16.0/mm^2^, *p* < 0.01) and amoeboid (RA: 11.1 ± 1.0 vs. OIR: 87.2 ± 13.4/mm^2^, *p* < 0.001) microglia were observed in OIR retinae when compared with RA retinae, while the numbers of ramified microglia were comparable between RA and OIR groups (RA: 76.8 ± 8.1 vs. OIR: 70.9 ± 9.8/mm^2^, *p* = 0.6669) (Figures 2I,J,K–M). In RA retinae, the association between retinal vessels and microglia was not observed (vascular meshes: *r* ^2^ = 0.0087, *p* = 0.7204; total vessel length: *r* ^2^ = 0.0259, *p* = 0.5829; Figure 2I”, Figures 7G,G’). In OIR retinae, the total length of retinal vessels was positively associated with the number of amoeboid microglia (*r* ^2^ = 0.3802, *p* = 0.0064) and with the number of total microglia (*r* ^2^ = 0.2297, *p* = 0.0442) (Figure 2J”, Figures 7H’,I’); however, this association was not found between the number of vascular meshes and the number of retinal microglia (amoeboid: *r* ^2^ = 0.2175, *p* = 0.0511; total: *r* ^2^ = 0.1741, *p* = 0.0849; Figure 2J”, Figures 7H,I).

In the mid-peripheral superficial retina, again, OIR retinae showed increased total (RA: 91.6 ± 12.1 vs. OIR: 152.1 ± 7.0/mm^2^, *p* < 0.01) and amoeboid (RA: 9.4 ± 5.6 vs. OIR: 72.1 ± 9.9/mm^2^, *p* < 0.001) microglia when compared with RA retinae, while the numbers of ramified microglia were comparable between two groups (RA: 82.2 ± 10.1 vs. OIR: 80.0 ± 6.3/mm^2^, *p* = 0.8513) (Figures 3J,K, L-N). In RA retinae, the total vessel length, but not the number of vascular meshes, was positively associated with the number of total microglia (vascular meshes: *r* ^2^ = 0.1124, *p* = 0.2044; total vessel length: *r* ^2^ = 0.6497, *p* = 0.0002; Figure 3J”, Figures 8G,G’). In OIR retinae, the retinal vessels were positively associated with the number of amoeboid microglia (vascular meshes: *r* ^2^ = 0.2075, p = 0.0130; total vessel length: *r* ^2^ = 0.1736, p = 0.0245; Figure 3K””, Figures 8H,H’); however, the associations were not found with the number of total microglia (vascular meshes: *r* ^2^ = 0.0397, p = 0.3000; total vessel length: *r* ^2^ = 0.0591, p = 0.2031; Figure 3K”, Figures 8I,I’).

In the central deep retina, OIR retinae showed a significant increase in ramified microglia in central areas (RA: 80.4 ± 9.8 vs. OIR: 118.3 ± 7.1/mm^2^, *p* < 0.05; Figures 4E,F,G). In RA retinae, the amount of retinal vessels was positively associated with the number of total microglia (vascular meshes: *r* ^2^ = 0.4221, *p* = 0.0019; total vessel length: *r* ^2^ = 0.6856, *p* < 0.0001; Figure 4E”, Figures 9E,E’). In OIR retinae, no association was observed between the amount of retinal vessels and retinal microglia number (vascular meshes: *r* ^2^ = 0.0195, *p* = 0.5256; total vessel length: *r* ^2^ = 0.0003, *p* = 0.9373; Figure 4F”, Figures 9F,F’).

In the mid-peripheral deep retina, an increasing trend of microglia number was observed in OIR retinae (RA: 89.5 ± 14.9 vs. OIR: 127.6 ± 11.7/mm^2^, *p* = 0.0716; Figures 5H,I,J). In RA retinae, the total vessel length was observed to be positively associated with the number of total microglia (*r* ^2^ = 0.2803, *p* = 0.0164), while the association was not found between the number of vascular meshes and the number of total microglia (*r* ^2^ = 0.1397, *p* = 0.1045) (Figure 5H”, Figures 10H,H’). In OIR retinae, no significant association was found between the retinal vessels and retinal microglia (vascular meshes: *r* ^2^ = 0.0765, *p* = 0.1256; total vessel length: *r* ^2^ = 0.0502, *p* = 0.2179) (Figure 5I”, Figures 10I,I’).

Taken together, at P30, the strong association between superficial retinal vasculature and amoeboid microglia in OIR was still observed. The positive association between deep retinal vessels and microglia was observed in RA but not in OIR retinae.

In summary, there was a positive association between deep retinal vasculature and microglia in RA. A positive association between superficial total vessel length (rather than number of meshes) and total microglia was also observed in RA at several time points. In OIR, there was a strong association between superficial (not deep) retinal vasculature and microglia. Microglial activation (reflected by increased amoeboid form and decreased ramified form) and proliferation (reflected by increased total number) may occur during OIR. These associations suggest a modulatory role of microglia in retinal vascular stability.

### 3.3 Microglial inhibition led to exacerbated retinal vascular damage in OIR retinae

**FIGURE 11:**
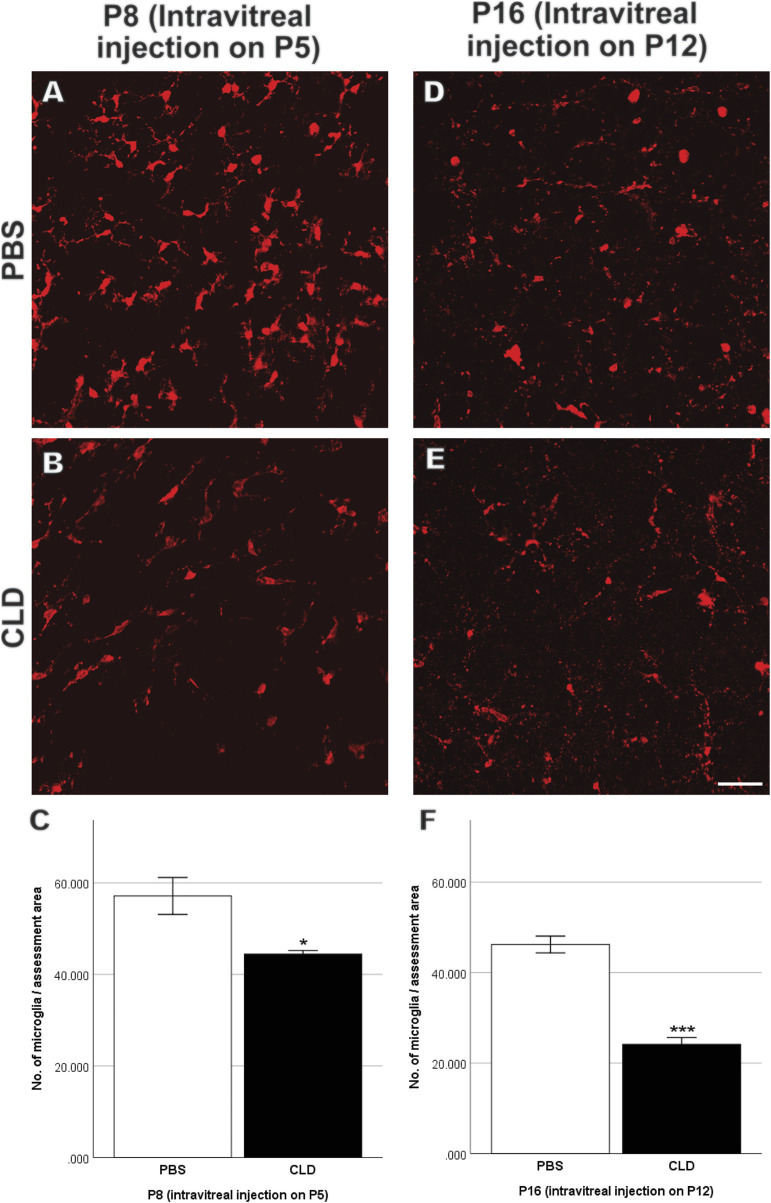
Retinal microglia quantitation in P8 and P16 retinae with intravitreal injection of liposomes-PBS/CLD on P5 and P12, respectively. Representative images of Iba-1-stained retinal whole mounts (red) are shown. Several amoeboid microglia among ramified ones were observed in both PBS- **(A,D)** and CLD-treated **(B,E)** retinae. The total number of microglia was significantly decreased in CLD-treated retinae compared with PBS-treated ones in both P8 and P16 retinae with intravitreal injection on P5 and P12, respectively **(C,F)**. n = 6–8 in each group, *p < 0.05, ***p < 0.001, compared with the PBS group, unpaired *t*-test. Scale bar, 50 μm.

**FIGURE 12:**
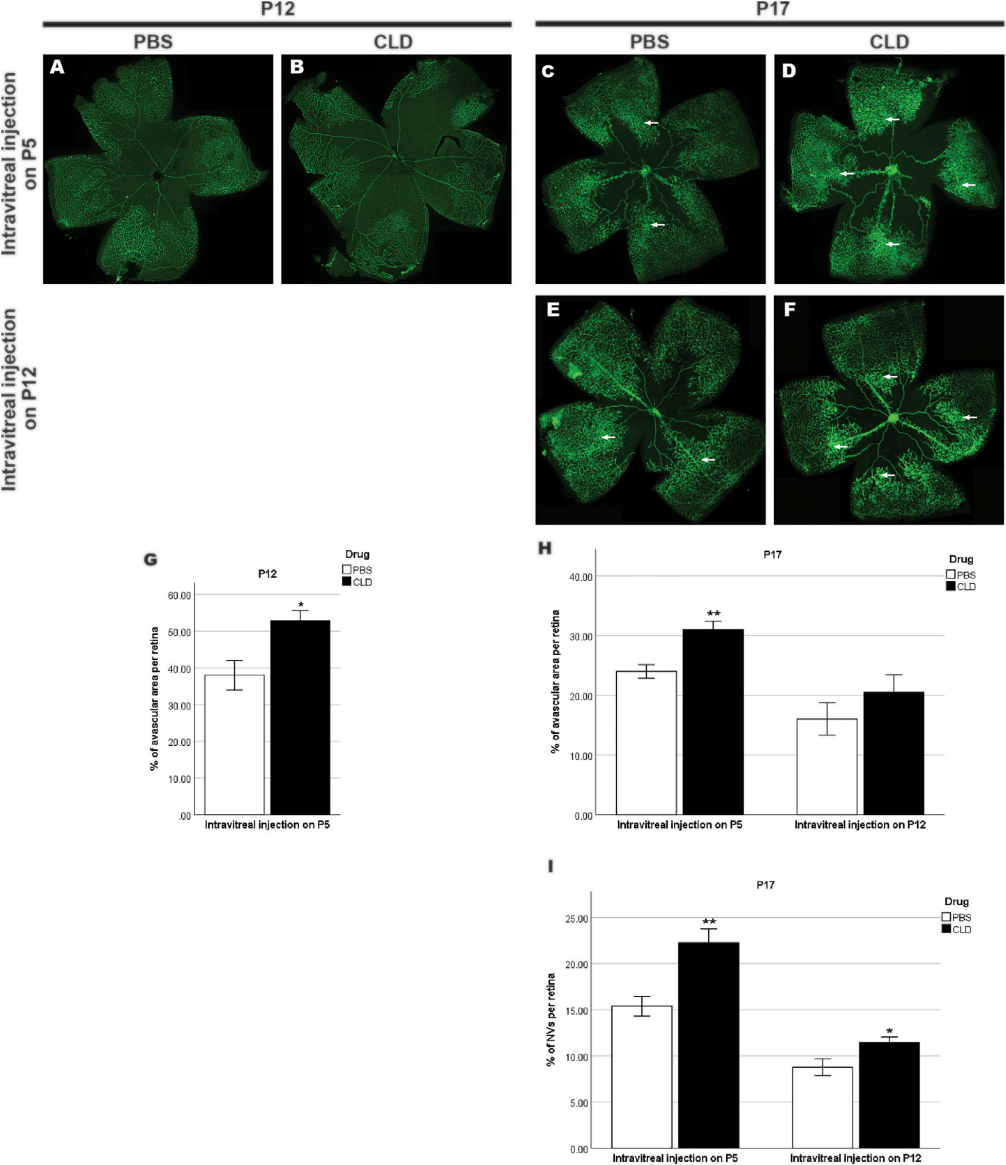
Retinal vasculature of OIR retinae with intravitreal injection of liposome-PBS/CLD. GS-IB4-stained retinal vasculature (green) in whole-mounted OIR retinae is shown. A central avascular zone could still be observed in both PBS- **(A,C,E)** and CLD-treated **(B,D,F)** P12 and P17 OIR retinae. The size of the avascular zone in CLD-treated OIR retinae was larger than that of PBS-treated OIR retinae on P12 and P17 with intravitreal injection on P5, whereas this increase was not significant on P17 OIR retinae with intravitreal injection on P12 **(G,H)**. Neovascularizations (NVs, indicated by arrows) could be observed in the mid-peripheral sites of both P17 PBS- and CLD-treated OIR retinae **(C,D,E,F)**. CLD-treated OIR retinae showed more NVs compared with PBS-treated OIR retinae with intravitreal injection of CLD on P5 or P12 **(I)**. n = 6–8 in each group, *p < 0.05**p < 0.01, compared with the PBS group, unpaired *t*-test.

To further validate the role of microglia in retinal vascular pathology in OIR, we pharmaceutically reduced the number of microglia using CLD. When intravitreal injection of liposomes-PBS/CLD was performed on P5, a significant reduction in the number of microglia was observed in CLD-treated retinae when compared with PBS-treated retinae (PBS: 57.1 ± 4.0 vs. CLD: 44.4 ± 0.8/assessment area, Figures 11A–C) at P8. On P12, a central vaso-obliterated zone was observed in both PBS- and CLD-treated OIR retinae. CLD-treated OIR retinae showed a larger avascular zone than PBS-treated OIR retinae (PBS: 38.0 ± 4.0% vs. CLD: 52.3 ± 2.9% per retina, Figures 12A,B,G). On P17, the central vaso-obliterated zone could still be observed in central areas of OIR retinae. Retinae treated with CLD still showed increased size of the central avascular zone, compared with PBS-treated ones (PBS: 24.0 ± 1.1% vs. CLD: 31.0 ± 1.5% per retina, Figures 12C,D,H). NVs were observed in mid-peripheral OIR retinae and reached their peak on P17. More NVs were observed in CLD-treated OIR retinae when compared with PBS-treated OIR retinae (PBS: 15.4 ± 1.1% vs. CLD: 22.3 ± 1.5% per retina, Figures 12C,D,I).

In mice with liposomes-PBS/CLD injection on P12, CLD treatment led to around 50% reduction of microglia number in retinae compared with PBS treatment (PBS: 46.2 ± 1.8 vs. CLD: 24.2 ± 1.5/assessment area, Figures 11D–F) at P16. On P17, the difference in the size of the central avascular zone between OIR CLD and OIR PBS retinae was not significant (PBS: 16.0 ± 2.7% vs. CLD: 20.5 ± 2.9% per retina, Figures 12E,F,H). In the mid-peripheral areas of OIR CLD retinae, increased NVs were observed compared with those of OIR PBS retinae (PBS: 8.8 ± 0.9% vs. CLD: 11.5 ± 0.6% per retina, Figures 12E,F,I)

These observations indicated that inhibition of microglia by CLD during both hyperoxic and hypoxic OIR processes led to more severe retinal vascular damage.

## 4 Discussion

In this study, we profiled the time course of microglial status and its association with the retinal vascular pattern at physiological RA and pathological OIR conditions. In the superficial retina, retinal microglia had already activated during the hyperoxia treatment, indicated by the increase in the number of total and amoeboid microglia in P12 OIR retinae. This activation continued during the hypoxic stage of OIR, which peaked on P21. Activation of microglia was still present on P30 in our observation process. In the deep retina, an increased number of microglia was noted in P17 OIR retinae, and this increase in deep microglia number kept increasing by the end of observation on P30. In the deep layer of P17 OIR retinae, there was no obvious change in microglia number in the central retina. This finding was different from our previous report (Fu et al., 2012). The use of different mouse strains may account for this phenomenon. Previously, C57BL/6N mice were used, which contained a nonsense *Rd8* mutation of the *Crb1* gene leading to photoreceptor degeneration in the nasal inferior retinal region as early as 2 weeks after birth (Mehalow et al., 2003; Mattapallil et al., 2012; Mekada and Yoshiki 2021). Abnormal photoreceptor morphogenesis and function in C57BL/6N mouse retinae may result in increased microglial response in the deep retina, which was not observed in retinae with C57BL/6J background.

Activation of microglia during OIR has been noted in several studies. It has been reported that an increased number of microglia was observed in P17 and P21 OIR retinae (Davies et al., 2006). Increased microglia in the superficial central retina from P12 to P14 and from P16 to P18, but decreased microglia in the deep central retina from P12 to P14 were also reported (Fischer et al., 2011). Our previous study also showed microglia activation in P17 superficial OIR retinae (Fu et al., 2012). Increased microglia were observed in P18 OIR retinal sections, which were also associated with reduced deep retinal plexus (Vessey et al., 2011). The expression of inflammatory cytokines, most of which are secreted by microglia, were upregulated in the OIR retina as well, indicating the involvement of microglia in OIR (Zhang et al., 2009; Yang et al., 2016). These studies revealed some properties of microglial behavior during OIR. In our present study, we discriminated between superficial and deep retinal layer vessels and microglia using confocal microscopy, thus focusing on counting the microglia in the specific layer in the same visual field for assessment. Previous results were mainly based on observations in retinal whole mounts and sections under a plain microscope, or from molecular quantitation using biomarkers.

In our observations, amoeboid microglia were located in retinal areas where abnormal vascular changes occurred. In P12 OIR retinae, successive hyperoxia treatment disturbed the physiological local retinal hypoxia for retinal vasculature development (Fruttiger, 2007); normal retinal vessel growth was disrupted, which was replaced with the central vaso-obliterated vascular network. At this time, despite the lack of blood vessel irrigation, the ambient oxygen level was high enough to support the metabolic demand of retinal cells. Although the general homeostasis was maintained, microglia might have sensed the changes in the environment; thus, a mild increase in the amoeboid microglia was observed in both central and mid-peripheral superficial retinae of OIR-treated mice. The number of ramified microglia in superficial OIR retinae decreased simultaneously, indicating the transition of microglia from the resting form to an activated form. It is reported that under normal conditions, depletion of retinal microglia led to delayed retinal vascularization and reduced retinal vessel density in rats (Checchin et al., 2006). Microglia can facilitate retinal vascular maturation at the early stage of rodents’ life. Our microglia depletion study further supported these deductions, as a loss of microglia from P7 led to an enlarged vaso-obliteration area on P12. On P17, the central vaso-obliteration was still observed despite shrinkage in the area. In response to 5 days of relative hypoxia, activation of microglia was obvious in OIR retinae. In central superficial retinae, activation of microglia was necessary to rebalance the disruption of homeostasis due to hypoxia. At the same time in mid-peripheral retinae, not only revascularization but also NVs occurred. Microglia might participate in the phagocytosis of NVs to initiate the regression process of the NV tufts. In non-tuft areas of P17 OIR retinae, not only amoeboid microglia but also ramified ones increased. It is possible that microglia in non-tuft areas proliferated in response to the need for microglia in adjacent areas with NVs, while increased activated microglia were about to migrate to the hypoxic or neovascularized areas nearby to function as phagocytes. With the progression of NV regression, more activated microglia were required to participate in removing NV tufts; it is, therefore, reasonable to observe the increase of activated microglia that peaked on P21. After P21, the microglia in the superficial retina remained activated, but to a smaller extent, indicating their return to the resting condition.

As NVs completely regressed starting P21, OIR retinae were almost fully covered by retinal vessels with no observable NV tufts. The retinal vessels were then compared between RA and OIR retinae by analyzing the total length of the vessels and the number of vascular meshes formed in each assessment region. The total length of the vessels reflected the amount of the retinal vessels, while the number of vascular meshes reflected the complexity of the retinal vascular network.

During the development of vascular meshwork, the blood vessels undergo a series of pruning and remodeling processes. The number of vessels and vascular meshes gradually decreases during development so that the irrigated areas receive enough blood supply but with fewer blood vessels; this serves to increase the efficiency of blood flow (Chen et al., 2012). In our present study, during the examination period from P21 until P30, the total length of the vessels and the number of vascular meshes decreased gradually in each assessment region in both RA and OIR retinae, indicating that OIR retinal vessels also underwent a process of re-organization to enhance the blood flow efficiency. In the OIR model, increased superficial retinal vessels might attribute to hypoxia-induced NVs. Hypoxia induced drastic proliferation of vascular endothelial cells to compensate for the impact of insufficient oxygen supply. It is reasonable that the increased vascular meshwork supplied the hypoxic area with blood flow, but the organization of the vascular network was not ideal. Therefore, the increased microglia, most of which were activated ones, might contribute to help re-organize the retinal vascular architecture to make it simplified. Furthermore, the positive association between microglia and retinal vessels was noticed in all P21, P25, and P30 OIR retinae that were assessed, indicating that microglia participate in the retinal vasculature re-organization process. On the other hand, this phenomenon showed that retinal vasculature was still abnormal in OIR retinae, even when NVs totally disappeared and the whole retina was completely covered by retinal vessels, according to the differences in retinal vessel parameters between two groups. Based on our results, the amount of microglia in the superficial retina could be used to reflect the retinal vascular conditions in late OIR; at the same time, the number of retinal vessels might also be used to indicate the microglia behavior in late OIR.

Interestingly, in the deep retina, the trend of vascular changes during late OIR was opposite to that in the superficial retina. The total length of vessels and the number of vascular meshes both decreased when compared with RA retinae since P17. The number of microglia was increased since P17, and these findings are similar to others’ observations (Fischer et al., 2011; Vessey et al., 2011). The number of microglia in the deep retina was negatively associated with the two indices of retinal vessels. Furthermore, most of the increased microglia are presented with a ramified morphology. It is already known that the photoreceptor function is compromised in ROP patients, which was also observed in ROP mice and rats (Akula et al., 2007; Akula et al., 2010; Hansen et al., 2017; Fu et al., 2018). Such neuronal changes also lead to vascular consequences. Normally, photoreceptors, especially rods, consume the most oxygen and energy and are almost entirely supported by choroidal circulation. However, the choroid is too thin to help photoreceptors counteract the impact of drastic hyperoxia or hypoxia shift; thus the demand for oxygen is switched to the inner retina, where deep vasculature locates (Hansen et al., 2017). We also observed reduced neuronal activities in mouse OIR retinae on P30 (Fu et al., 2015). It is plausible that the reduced photoreceptor metabolic demand led to delayed deep retinal vessel growth. It has been shown that photoreceptor glucose metabolism controls deep vascularization in mice modeling Phase I ROP (Fu et al., 2018). For unchanged microglial morphology in the deep retina, we speculate that the OIR process might just halt the normal development of the deep retinal vessels due to altered photoreceptor metabolism. Unlike the superficial retina, the deep retina did not experience the vaso-obliteration process, and the regional vascular development was just delayed but not interrupted; the change in local homeostasis was not drastic enough to induce microglia reactivity. Microglia continued to guide the formation of deep retinal vessel plexus, while presented with normal morphology. Further investigation on the role of microglia located in the deep retina during OIR is warranted.

We further applied an inhibitory strategy to test whether retinal microglia are beneficial or unfavorable for retinal vasculature in the mouse OIR model. The inhibition of retinal microglia was achieved by intravitreal injection of liposomes-CLD, which specifically targets retinal microglia. The injections were performed at two different time points, so that we could investigate microglia’s role at different stages of the OIR model. Pre-hyperoxic inhibition helped determine microglia’s role during vaso-obliteration, and post-hyperoxic inhibition facilitated our understanding of microglia’s character in the proliferative stage of OIR.

Inhibition or depletion of macrophages/microglia can be achieved by several methods. Liposomes-CLD can be used to induce “macrophage suicide,” attributing to the phagocytic properties of monocytes. Liposomes-CLD has been widely used for the depletion of systemic macrophages in the studies of liver and lung diseases (Eichinger et al., 2015; Griesmann et al., 2016). Minocycline, a tetracycline derivative, is another commonly used inhibitor for microglia. It can inhibit the activation and proliferation of microglia by preventing the increase of phosphor-38 MAPK expression upon challenge (Tikka et al., 2001). Recent studies also showed that minocycline inhibits M1 polarization of microglia upon LPS challenge via inhibiting the NF-κB pathway (Kobayashi et al., 2013). With the development of genetic modification techniques, mice with deficiency in microglia function are available, for instance, the Cx3cr1-deficient mice (Zhao et al., 2009). However, the limitations of these strategies for microglia inhibition are also noticeable. The liposomes-CLD’s effects are transient for most purposes, and thus it needs to be injected repeatedly. Meanwhile, the systemic application of liposomes-CLD leads to inactivation of macrophages/microglia throughout the body, which may result in general side effects. The *in vivo* application of minocycline is also systemic; furthermore, due to its short half-life *in vivo*, minocycline needs to be injected twice a day, and the injections should be maintained throughout the experimental process. Frequent injections are not ideal for the vulnerable OIR-challenged neonatal mice. For transgenic mice with functionally modified microglia, the depletive effect is again systemic; therefore, microglia inhibition based on these methods is still not specific for retinal microglial inhibition.

In our present study, we applied a single injection of liposomes-CLD directly to the vitreous cavity. The CLD treatment led to a significant reduction in the number of Iba-1-positive cells in the superficial layer of the retina, compared with the PBS-treated ones. It has been reported that the effects of liposomes-CLD could last at least for 5 days (van Rooijen and Sanders 1994); therefore, the inhibitory effects of a single injection of liposomes-CLD fulfilled our experimental requirements. The effective period falls into the time window of hyperoxic treatment and proliferative stage of the mouse OIR model. Thus, we could discriminate the potential differences in microglia’s role at different stages of the OIR process. Furthermore, our strategy avoided other side effects related to systemic macrophage/microglia inhibition, such as suppression of necessary inflammatory responses and synaptic maturation during development.

Pre-hyperoxic inhibition of microglia gave clues on their role in the vaso-obliterative stage of OIR. Most of the studies on OIR focused on the NVs, and the interventions were often applied during the hypoxic phase of the OIR model, which resembles the situation in human beings at clinic. For the hyperoxia stage, intervention or prevention was seldom reported. Our experimental observations indicated that retinal microglia are essential for retinal vasculature maintenance in OIR. It has been reported that retinal microglia are involved in retinal vessel formation. The depletion of retinal microglia led to delayed retinal vessel development and decreased vascular density (Checchin et al., 2006; Zhang et al., 2012). Therefore, relatively immature retinal vessel organization might be more vulnerable to hyperoxic insult, and thus increased avascular zone was observed in microglia-inhibited OIR retinae. Tremblay et al. (2013) treated neonatal mice with LPS to induce inflammation. They found that retinal microglia were increased upon LPS challenge, and the vascular density was also increased. These findings showed that these microglia promoted retinal vessel growth. Our findings are partly consistent with these observations. An increased avascular zone caused more demands in oxygen consumption, thus leading to more NVs as a reasonable consequence as observed on P17.

Post-hyperoxic inhibition of microglia also led to increased NVs, indicating that microglia might also be important for retinal vascular organization during the proliferative stage of OIR. Macrophages/microglia are well known for their phagocytic capability. Microglia are postulated to be involved in clearance of the neovascularized tufts during the proliferative stage of OIR, by engulfing the NV tufts and inducing apoptosis in the endothelial cells of these abnormal vascular structures (Davies et al., 2008). Intravitreally injected myeloid progenitor cells finally differentiated into microglia and helped reduce the NVs in the mouse OIR model (Ritter et al., 2006). These microglia might cooperate with astrocytes, promoted the regrowth of blood vessels after vaso-obliteration in OIR, and thus resulted in less NVs afterward in the mouse OIR model, via VEGF and bFGF (Dorrell et al., 2010). These observations also support the idea that microglia may be beneficial for the retinal vasculature during OIR.

An inevitable issue on microglia in the OIR model is the source of the Iba-1-positive cells. Sharing almost the same cell markers, macrophages and microglia are indistinguishable under activated conditions. In the OIR model, the BRB is broken down and macrophages can then infiltrate into the retina, which has been demonstrated by detecting GFP-positive macrophages in the retinae of OIR mice that received intraperitoneal injection of macrophages of GFP mice (Kataoka et al., 2011). It has been noted that vitreous macrophages are adjacent to the NV tufts in the mouse OIR model, and they are capable of secreting VEGF, which appeared to contribute to the neoangiogenesis in this model (Naug et al., 2000). Several studies have shown that macrophages contribute to the NVs in OIR. Systemic application of liposomes-CLD led to macrophage depletion, which further resulted in reduced NVs in the mouse OIR model (Gao et al., 2016). Zhou et al. (2015) found that intravitreal injection of M2-polarized macrophages promoted NVs in the mouse OIR model. These macrophages might be detrimental to retinal vasculature during the OIR model. However, retinal microglia’s role might be different. As mentioned previously, intravitreally injected myeloid progenitor cells alleviated NVs in the OIR model, and they finally differentiated into microglia (Ritter et al., 2006). In developing neonatal mice, resident retinal myeloid cells may suppress the angiogenesis via a non-canonical Wnt-Flt1 pathway (Stefater et al., 2011). In the mouse OIR model, BALB/c mice showed comparable vaso-obliteration as C57BL/6J mice did, but almost no pathological angiogenesis on P17. One interesting reason is that BALB/c mice have more retinal microglia when compared with C57BL/6J mice. Increased microglia might contribute to the prevention of NV genesis or clearance of NVs (Ritter et al., 2006). A recent study using Cx3cr1^CreERT2^:Rosa26-tdTomato mice showed that increased Iba1+ cells in retinal NVs on P17 were predominantly local microglial cells but not infiltrated macrophages from the periphery. Furthermore, in the neovascular area on P17, microglia is about 96.1% of the myeloid cell population, and infiltrating macrophage is about 3.9%. Neglectable infiltrating macrophages are detected in the peripheral and vaso-obliterated region on P12. These cells showed upregulated mRNA levels for chemokine signaling, which would be probably responsible for microglial activation and migration. The levels of pro-angiogenic factors for NVs, such as *Epo*, *Ang2*, or *Vegf*, were not upregulated, indicating that these increased microglial cells in NVs were not contributing to the NV formation (Boeck et al., 2020). These findings may help explain the beneficial role of retinal microglia in OIR, in line with our observations; we speculated that resident microglia play a predominant role in regulating the vasculature in OIR retinae.

It is well documented that microglia activation is dynamic and can be categorized into M1 proinflammatory and M2 anti-inflammatory types, which are featured by different cytokines (Orihuela et al., 2016). It is of importance to fully characterize their function type in OIR retinae. Existing routine ways using co-staining of inflammatory markers with a microglial marker or single-cell transcriptomics have their own limitations. For immunostaining of secreted inflammatory markers, it cannot specify the source of the signals, as other retinal cells may secrete the same cytokines. For single-cell transcriptomics, the gene markers that distinguish resting and activated microglia at different locations are still to be determined at present. Recently developed multiplexed error-robust fluorescence *in situ* hybridization (MERFISH) might help resolve the source of the signal issue in retina research (Chen et al., 2015; Ferdous et al., 2022); it allows spatially resolved gene expression profiling of single cells in intact biological samples. It will be of great interest to apply this technique to the OIR study to help elucidate the exact role of microglia in the near future.

Based on our observations, retinal microglia might play a protective role in retinal vascular maintenance during the OIR process, as loss of microglia led to more severe vascular damage after hyperoxic insult in the mouse OIR model. Further investigations will be needed to explore the mechanisms behind microglial protection against OIR at different disease stages.

## 5 Conclusion

In the present study, we elaborated our knowledge of microglia response during the whole process of the mouse OIR model, by quantifying them in different retinal areas at different time points, with a clarification of their activation status. The activation of microglia was associated with vascular abnormalities throughout the whole OIR process. The microglia in superficial and deep retinal areas displayed different patterns in response to retinal vascular changes. Moreover, the activation of these cells lasted for a long time, even after the retinal vasculature appeared normal. Pharmacological inhibition of microglia during both hyperoxic and hypoxic stages of OIR led to exacerbated vascular damage. Therefore, retinal microglia might play a protective role in retinal vasculature maintenance during OIR. Targeting microglial activation may prevent and treat ROP at early and late stages.

## Data Availability

The raw data supporting the conclusion of this article will be made available by the authors, without undue reservation.
